# Supplementary material for: Short tandem repeat stutter model inferred from direct measurement of in vitro stutter noise
Source: Nucleic Acids Res. 2019 Jan 30;47(5):2436–45. doi: 10.1093/nar/gky1318 (PMC6412005; doi:10.1093/nar/gky1318)
Supplement: Supplementary Data [file gky1318_supplemental_files.zip › Short_Tandem_Repeat_stutter_model_inferred_from_direct_measurement_of_in_vitro_stutter_noise_-_Supplemental_Information.pdf]

# Supplemental Information

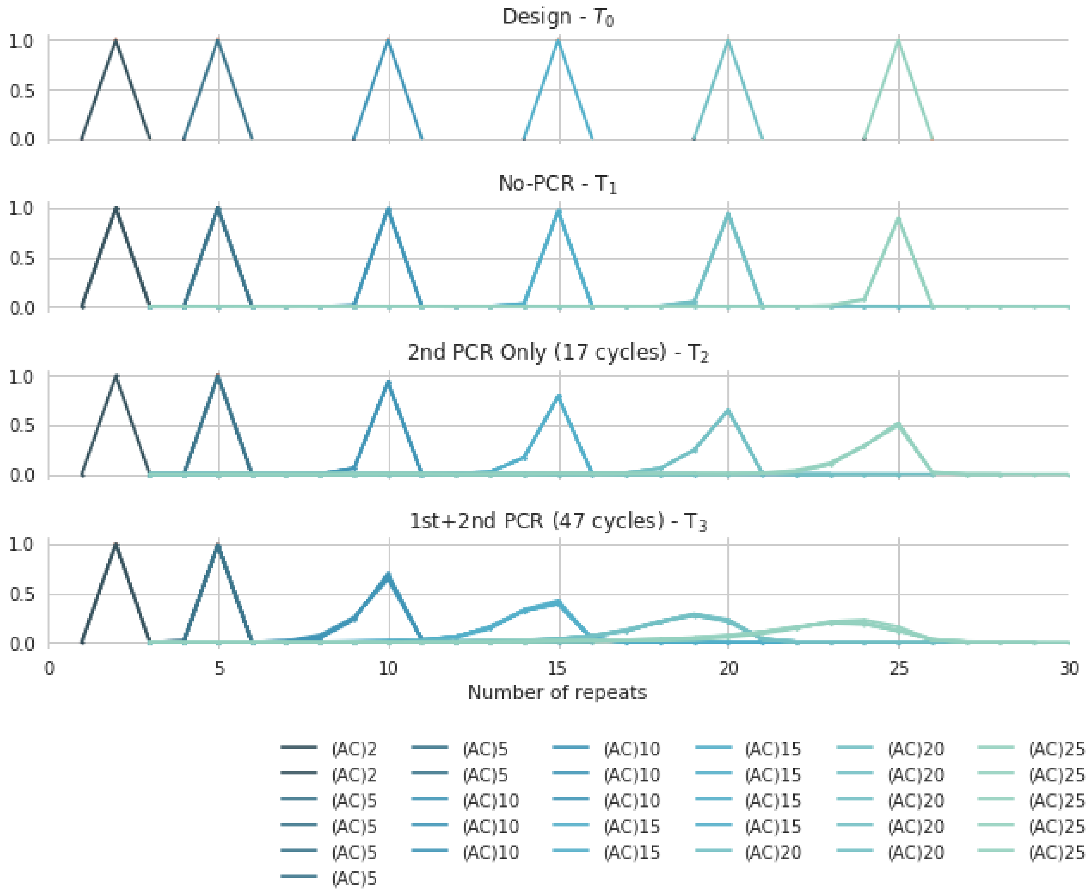


**Supplementary Figure S1. AC STRs repeat-number histograms, as were interpreted from sequencing results ($T_1$, $T_2$ and $T_3$), compared to their expected length, $T_0$ (designed sequence).**

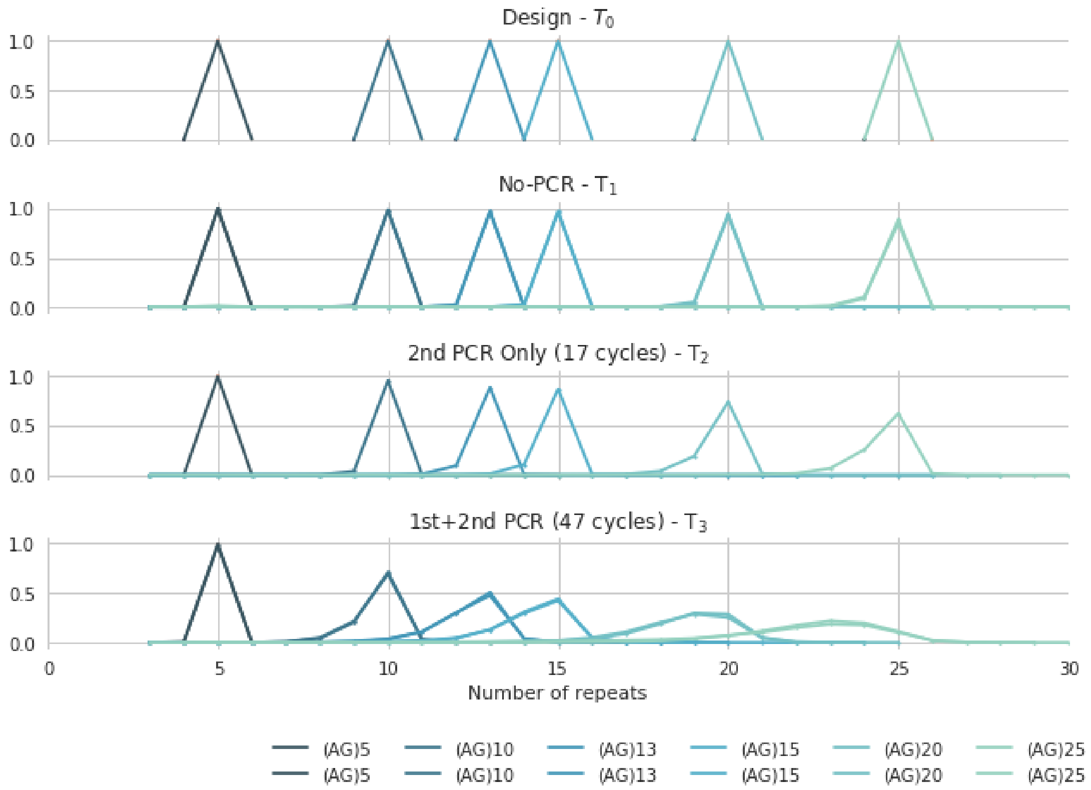


**Supplementary Figure S2. AG STRs repeat-number histograms, as were interpreted from sequencing results ($T_1$, $T_2$ and $T_3$), compared to their expected length, $T_0$ (designed sequence).**

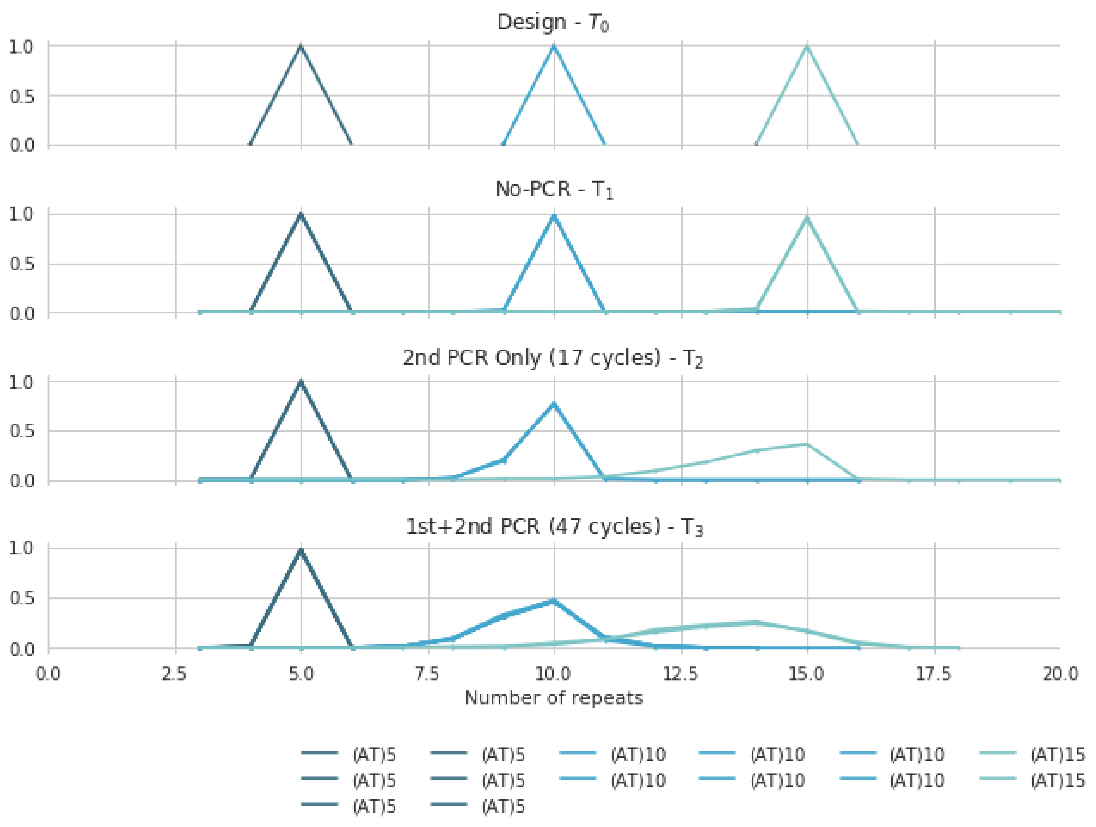


**Supplementary Figure S3. AT STRs repeat-number histograms, as were interpreted from sequencing results ($T_1$, $T_2$ and $T_3$), compared to their expected length, $T_0$ (designed sequence).**

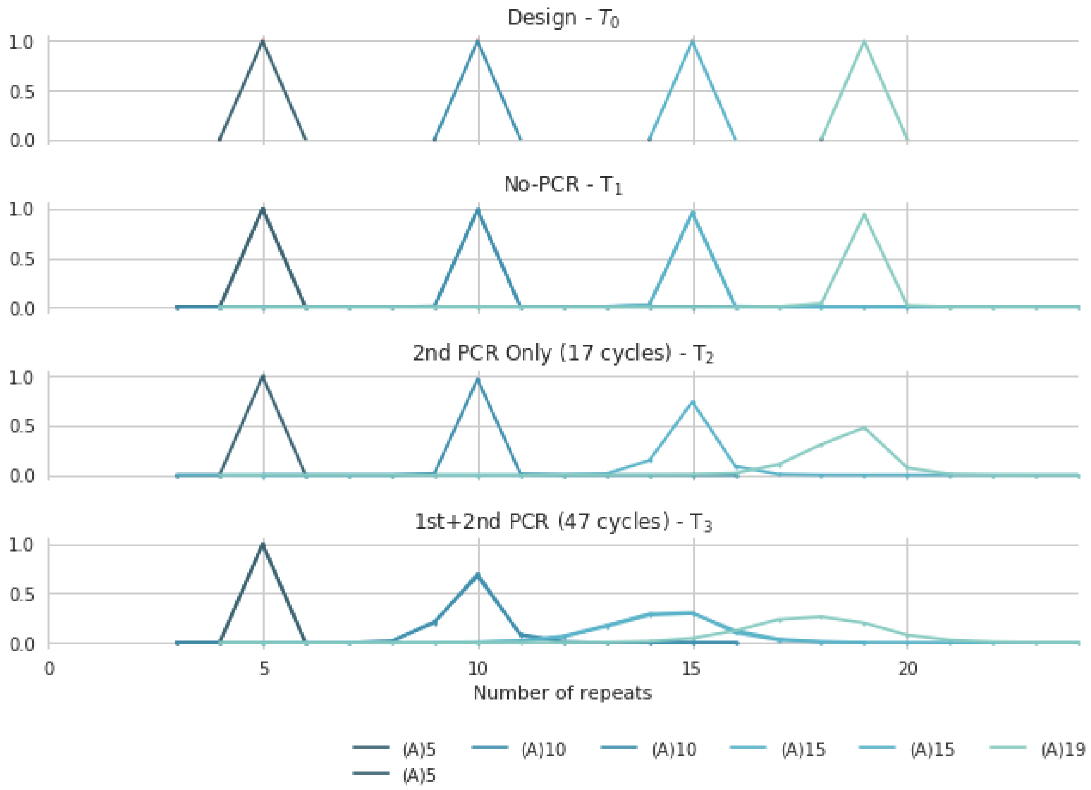


**Supplementary Figure S4. polyA STRs repeat-number histograms, as were interpreted from sequencing results ($T_1$, $T_2$ and $T_3$), compared to their expected length, $T_0$ (designed sequence).**

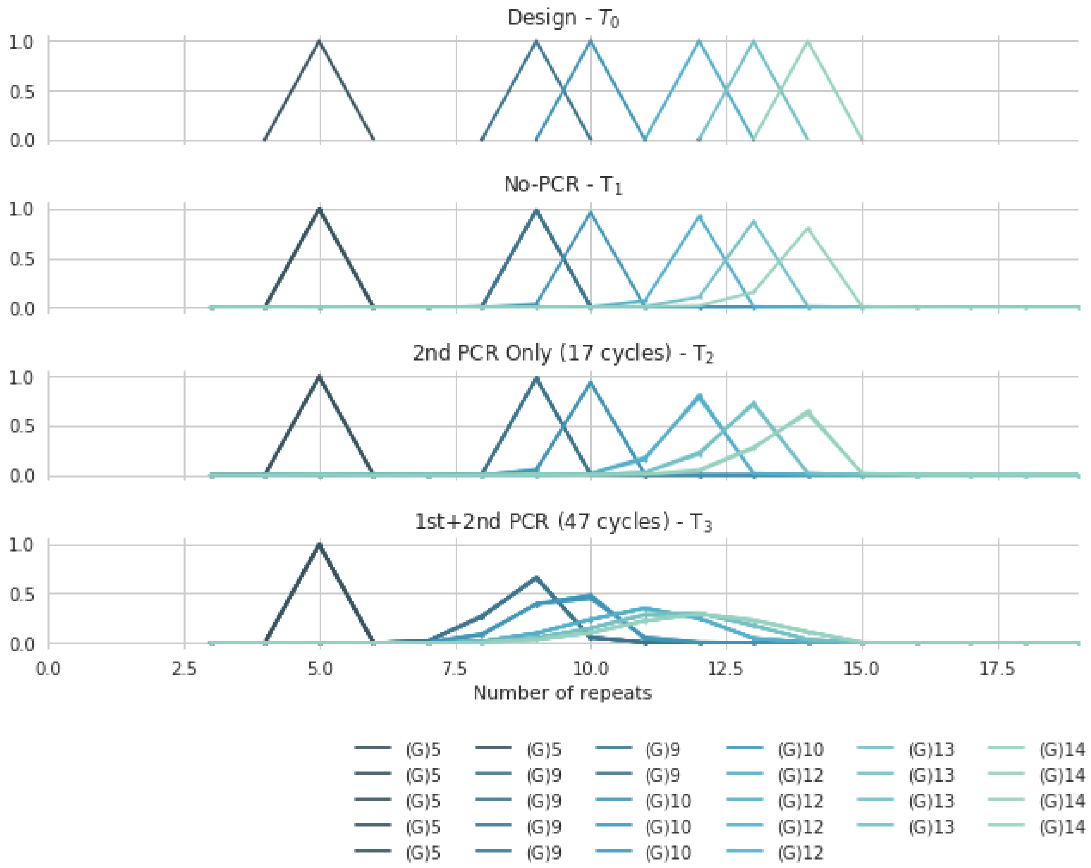


**Supplementary Figure S5. polyG STRs repeat-number histograms, as were interpreted from sequencing results ($T_1$, $T_2$ and $T_3$), compared to their expected length, $T_0$ (designed sequence).**

[Supplemental_Figure_S6_AC_series.pdf]

**Supplemental Figure S6. Depiction of fitted models for repeat unit type AC.** Each row shows a model type after calibration for the measured dataset. Left: Measured stutter histograms following the second PCR step in blue, all measurements (including all synthesized repeats and technical replicates) are overlaid. Best fitted model for each measured histogram is overlaid in green. Right: Theoretical space of the model histograms calculated for various lengths, simulated at 45 PCR cycles.

[Supplemental_Figure_S7_AG_series.pdf]

**Supplemental Figure S7. Depiction of fitted models for repeat unit type AG**. Each row shows a model type after calibration for the measured dataset. Left: Measured stutter histograms following the second PCR step in blue, all measurements (including all synthesized repeats and technical replicates) are overlaid. Best fitted model for each measured histogram is overlaid in green. Right: Theoretical space of the model histograms calculated for various lengths, simulated at 45 PCR cycles.

[Supplemental_Figure_S8_AT_series.pdf]

**Supplemental Figure S8. Depiction of fitted models for repeat unit type AT**. Each row shows a model type after calibration for the measured dataset. Left: Measured stutter histograms following the second PCR step in blue, all measurements (including all synthesized repeats and technical replicates) are overlaid. Best fitted model for each measured histogram is overlaid in green. Right: Theoretical space of the model histograms calculated for various lengths, simulated at 45 PCR cycles.

[Supplemental_Figure_S9_A_series.pdf]

**Supplemental Figure S9. Depiction of fitted models for repeat unit type A**. Each row shows a model type after calibration for the measured dataset. Left: Measured stutter histograms following the second PCR step in blue, all measurements (including all synthesized repeats and technical replicates) are overlaid. Best fitted model for each measured histogram is overlaid in green. Right: Theoretical space of the model histograms calculated for various lengths, simulated at 45 PCR cycles.

[Supplemental_Figure_S10_G_series.pdf]

**Supplemental Figure S10. Depiction of fitted models for repeat unit type G**. Each row shows a model type after calibration for the measured dataset. Left: Measured stutter histograms following the second PCR step in blue, all measurements (including all synthesized repeats and technical replicates) are overlaid. Best fitted model for each measured histogram is overlaid in green. Right: Theoretical space of the model histograms calculated for various lengths, simulated at 45 PCR cycles.

## Correlation

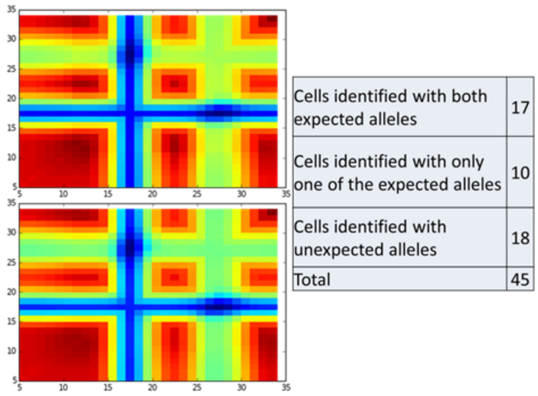

| | |
|---|---|
| Cells identified with both expected alleles | 17 |
| Cells identified with only one of the expected alleles | 10 |
| Cells identified with unexpected alleles | 18 |
| Total | 45 |

## Chi Square

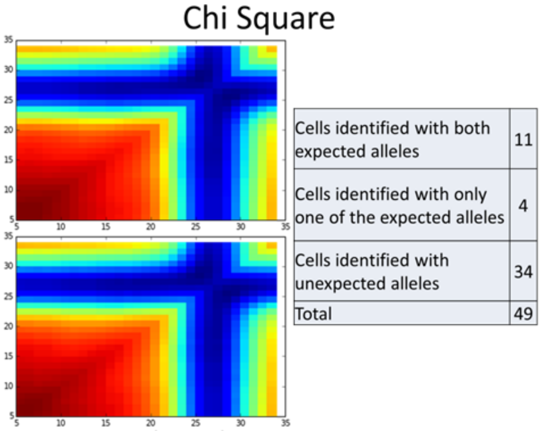

| | |
|---|---|
| Cells identified with both expected alleles | 11 |
| Cells identified with only one of the expected alleles | 4 |
| Cells identified with unexpected alleles | 34 |
| Total | 49 |

## Probability

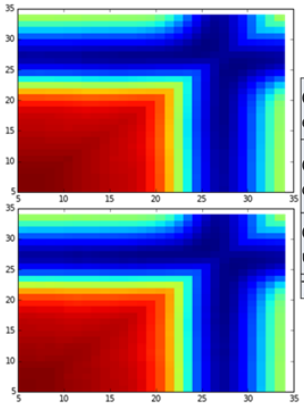

| | |
|---|---|
| Cells identified with both expected alleles | 0 |
| Cells identified with only one of the expected alleles | 0 |
| Cells identified with unexpected alleles | 49 |
| Total | 49 |

## Two-sample Kolmogorov–Smirnov test

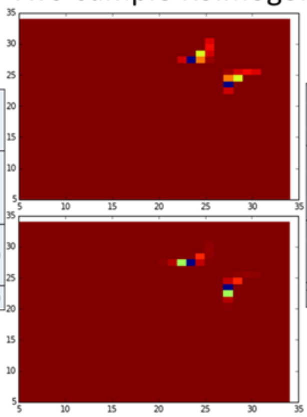

| | |
|---|---|
| Cells identified with both expected alleles | 0 |
| Cells identified with only one of the expected alleles | 0 |
| Cells identified with unexpected alleles | 49 |
| Total | 49 |

## Earth Movers Distance

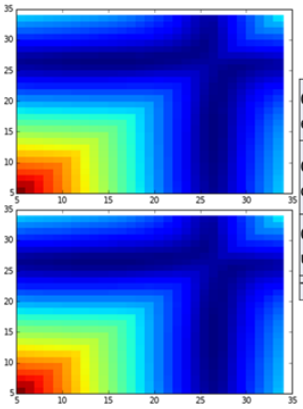

| | |
|---|---|
| Cells identified with both expected alleles | 0 |
| Cells identified with only one of the expected alleles | 0 |
| Cells identified with unexpected alleles | 49 |
| Total | 49 |

## Subtraction

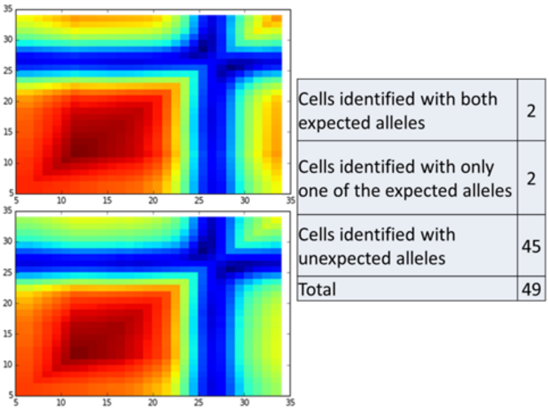

| | |
|---|---|
| Cells identified with both expected alleles | 2 |
| Cells identified with only one of the expected alleles | 2 |
| Cells identified with unexpected alleles | 45 |
| Total | 49 |

**Supplemental Figure S11. Comparison of distance metrics between histogram.** Example histogram sampled from NGS data of the H1 stem cell line, showing an AC locus from chromosome 9, with both alleles 25, 28 present at a 6/4 ratio. The histogram was measured against the space of possible model alleles. For each metric, a distances heatmap is plotted and compliance of to the single cell data is listed in the neighboring tables.

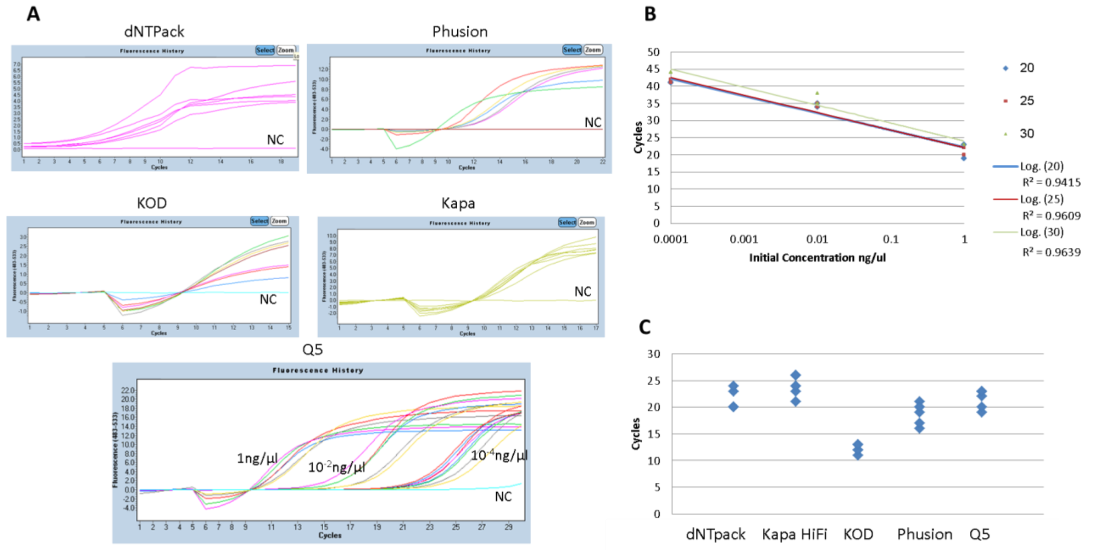


**Supplementary Figure S12. Validation of the R&B model utilizing enzyme comparison and serial dilution experiments.** STR plasmids $(AC)_{20}$, $(AC)_{25}$ and $(AC)_{30}$ were amplified in duplicates using different enzymes and different dilutions (Q5 enzyme only). A) Amplification curves for each enzyme. NC marks the negative control (water) and serial dilution curves (Q5 plot) are marked by their corresponding template concentration. B) Concentration accurate prediction from NGS datasets (Q5 serial dilution experiment). Each point in the graph represents a single PCR reaction dataset. x-axis shows the original template concentration (log scale). y-axis shows the calculated number of effective amplification cycles, as calculated from the model. The linear trend line ($0.94<R^2$) for each AC size reflects a successful prediction of the number of amplification cycles by the model. d) Calculated effective cycles for each PCR polymerase. Each column represents a different PCR polymerase enzyme, each with its calculated cycles dataset scattered on its y-axis (blue dots, not including $10^{-2}$ng/µl, $10^{-4}$ng/µl of Q5).

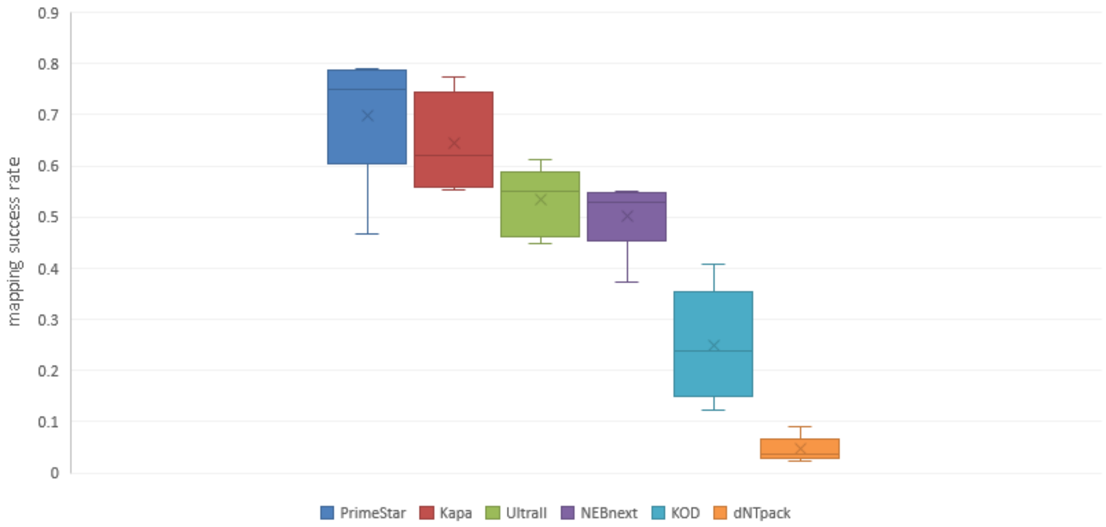


**Supplementary Figure S13. Preliminary polymerase enzyme experiment results**. Comparing the mapping rates for the NGS reads resulting from targeted amplification and barcoding PCR using various enzymes.

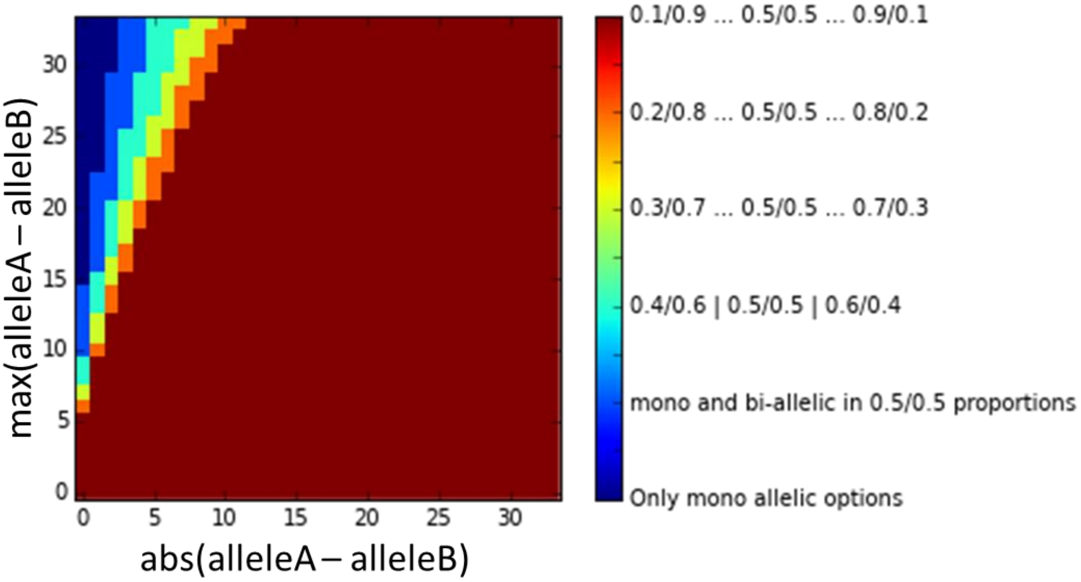


**Supplementary Figure S14. Limited space of biallelic proportions taken into account.** In order to minimize occurrences allele calling attributed to noise, we have limited the space of alleles and proportions that is being measured against the sequencing histograms.
