# Supplementary figures and images for: Short tandem repeat stutter model inferred from direct measurement of in vitro stutter noise
Source: Nucleic Acids Res. 2019 Jan 30;47(5):2436–45. doi: 10.1093/nar/gky1318 (PMC6412005; doi:10.1093/nar/gky1318)

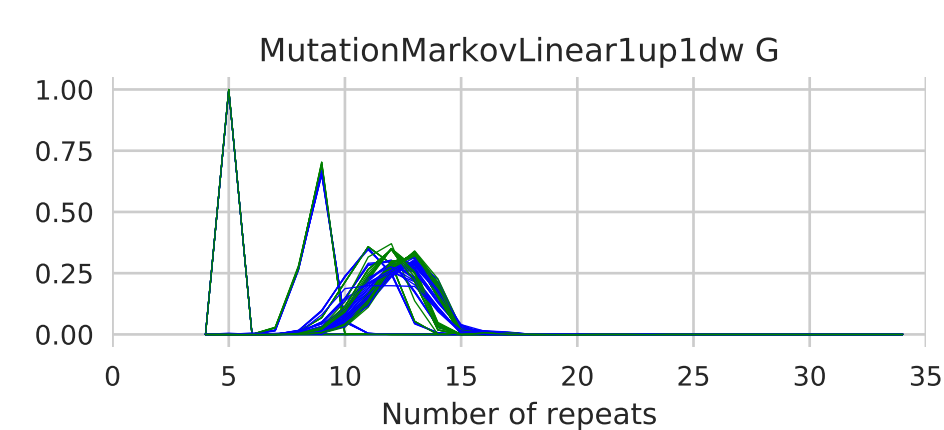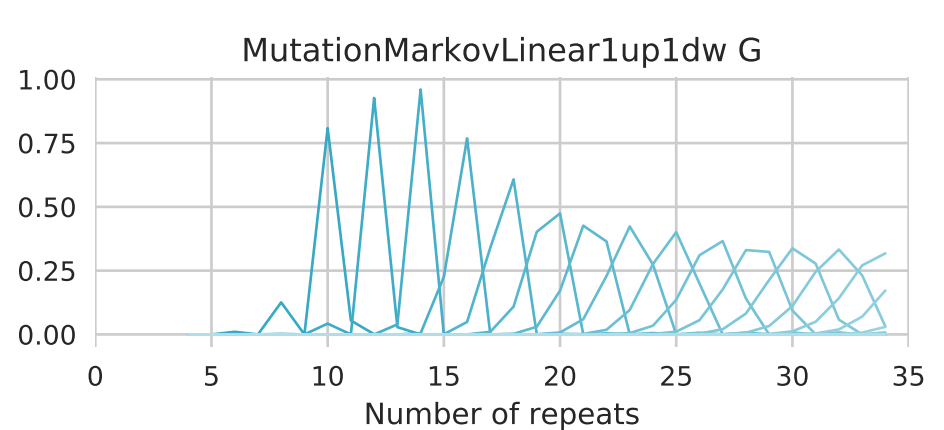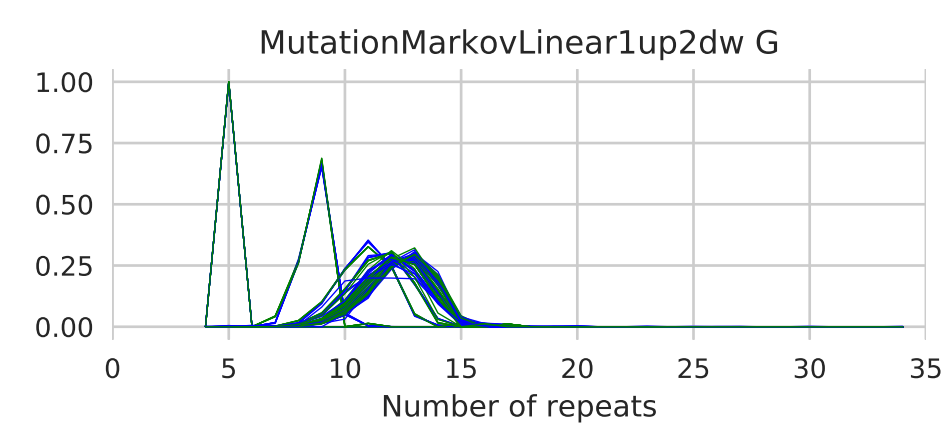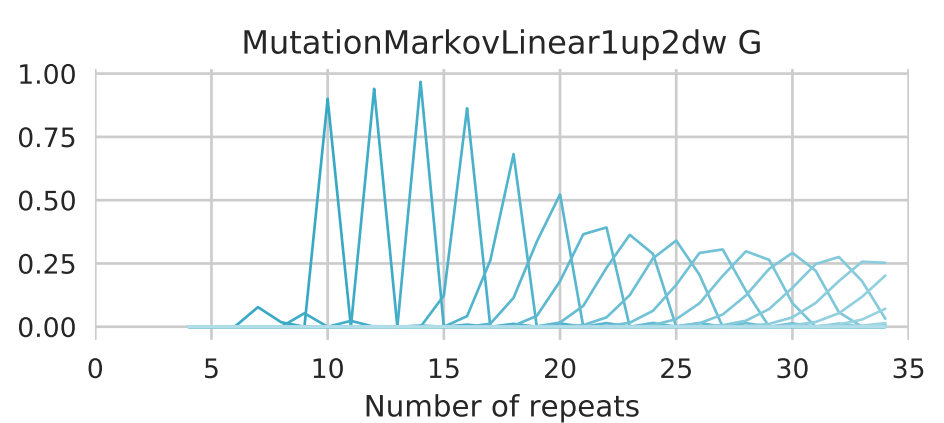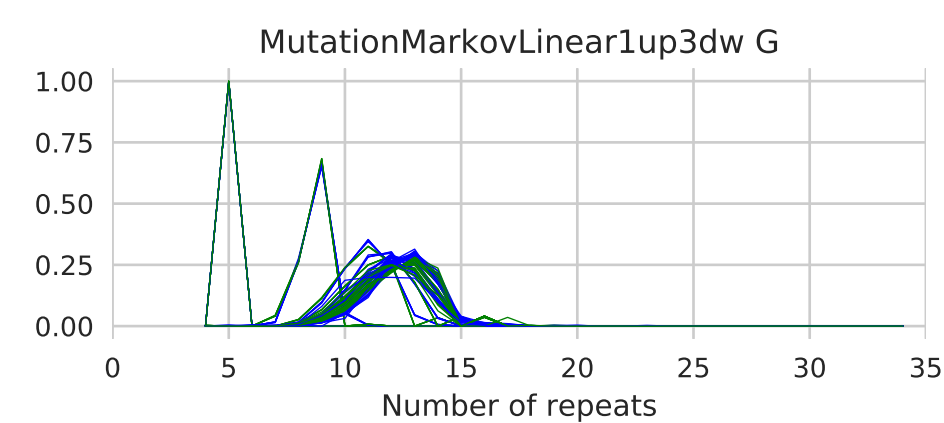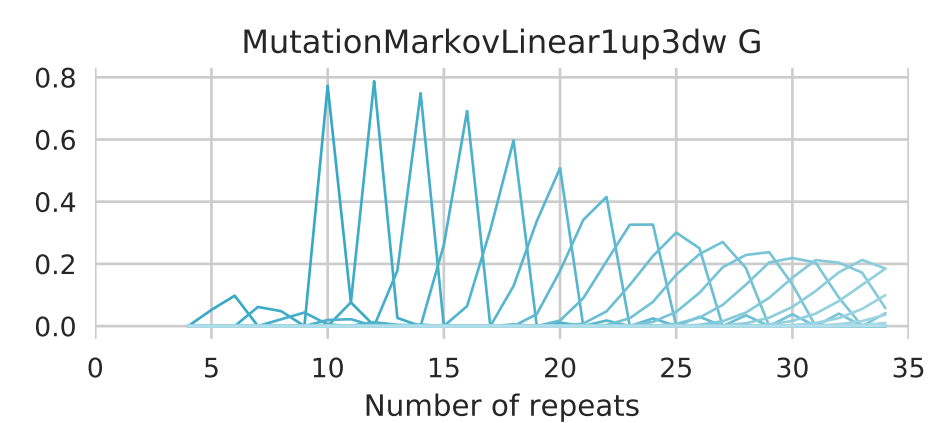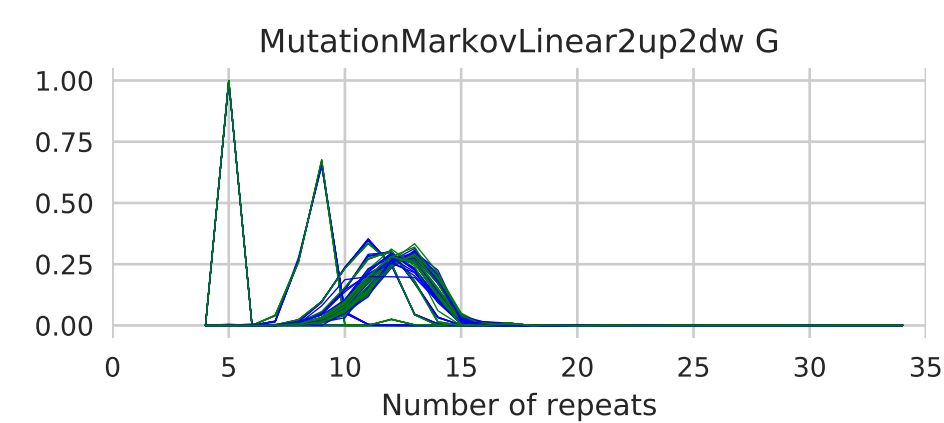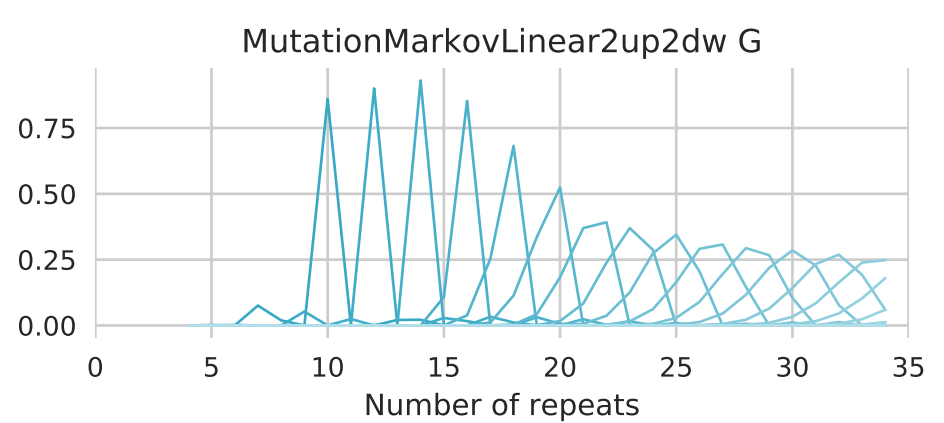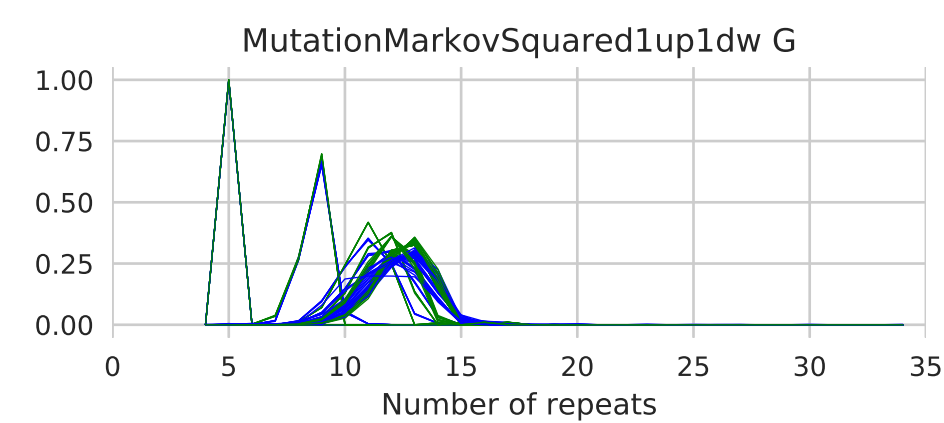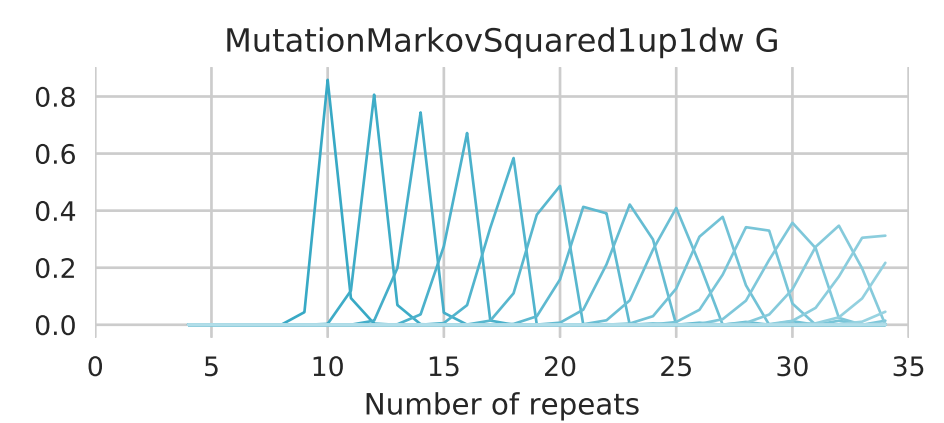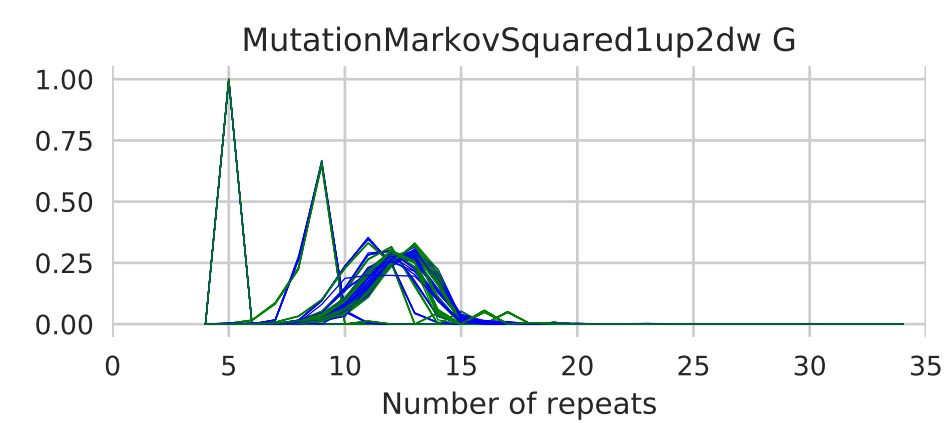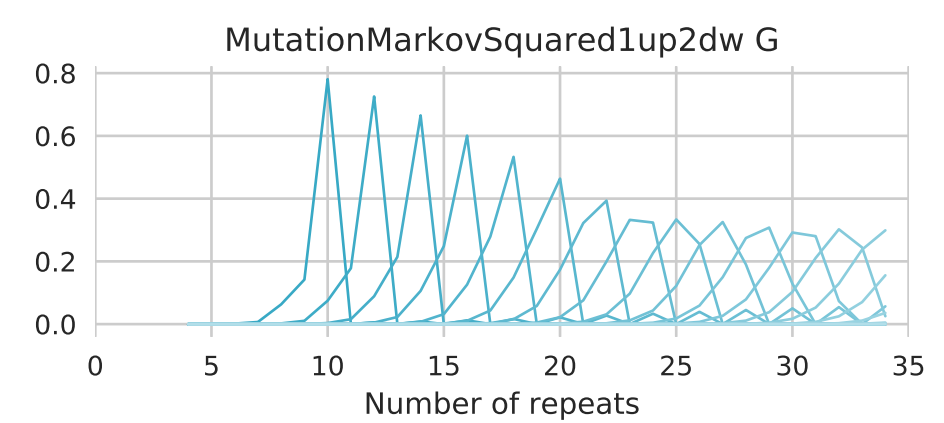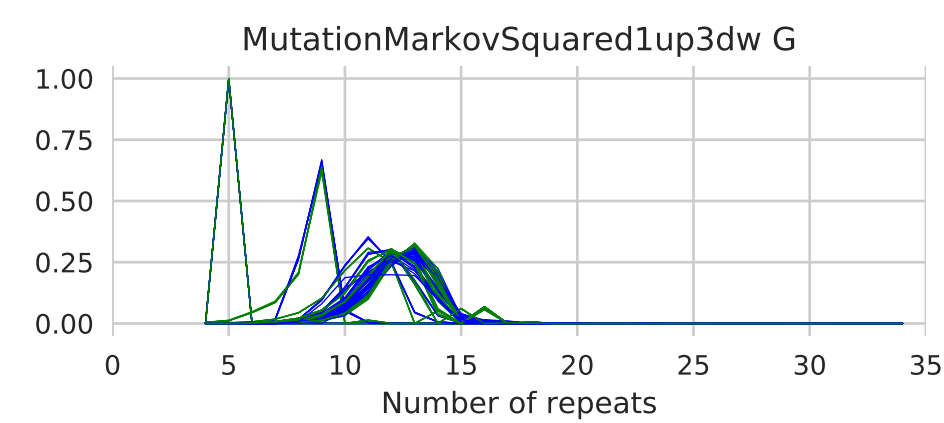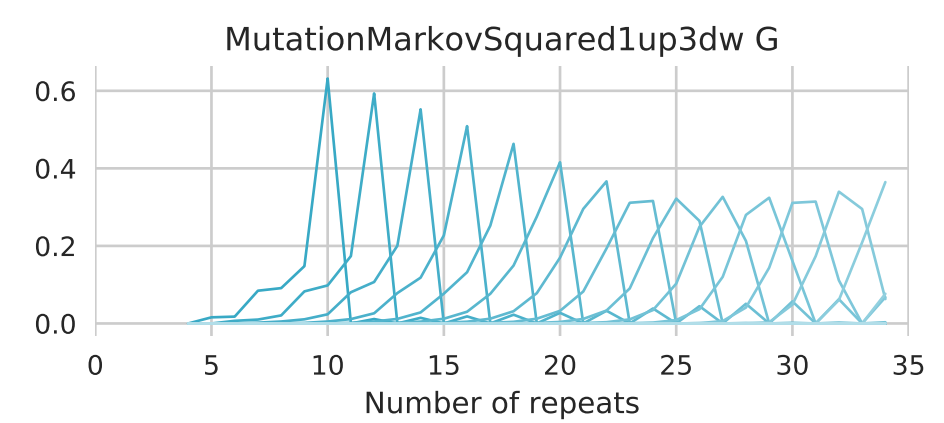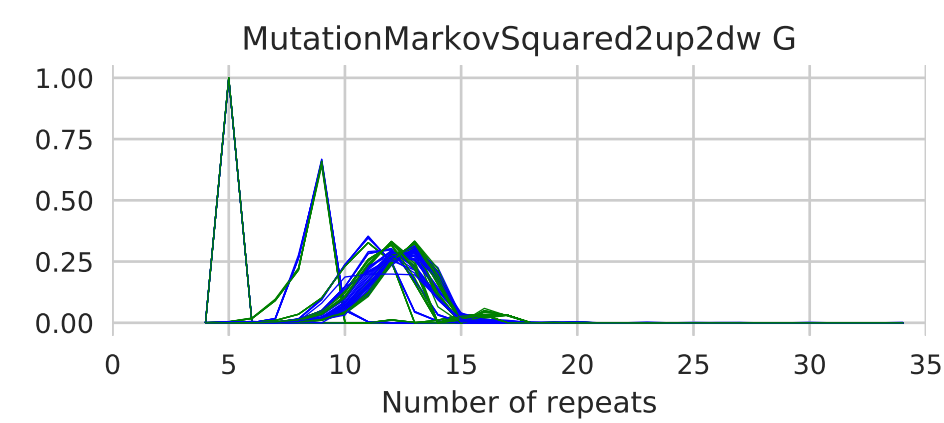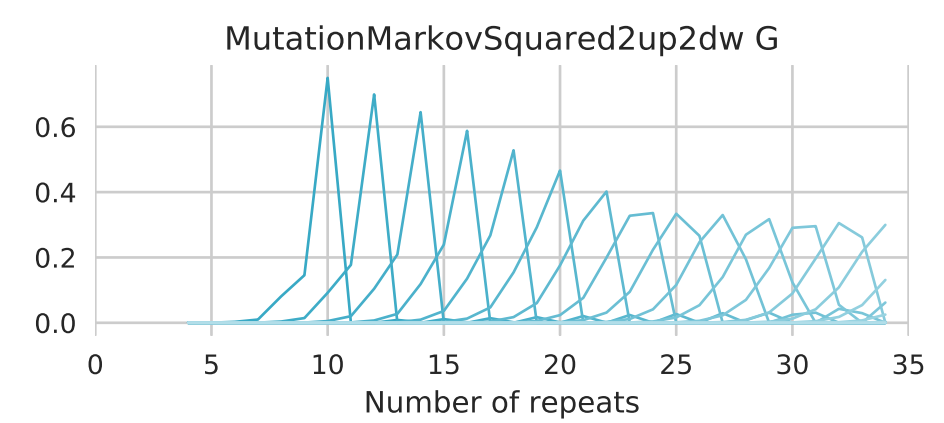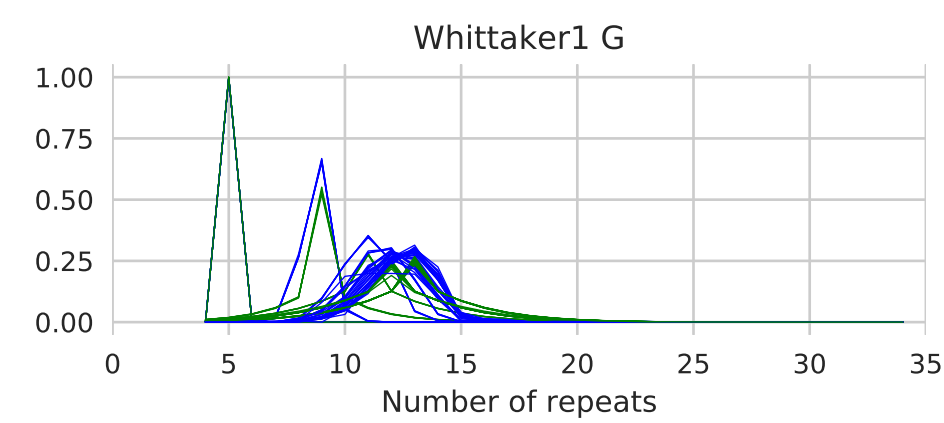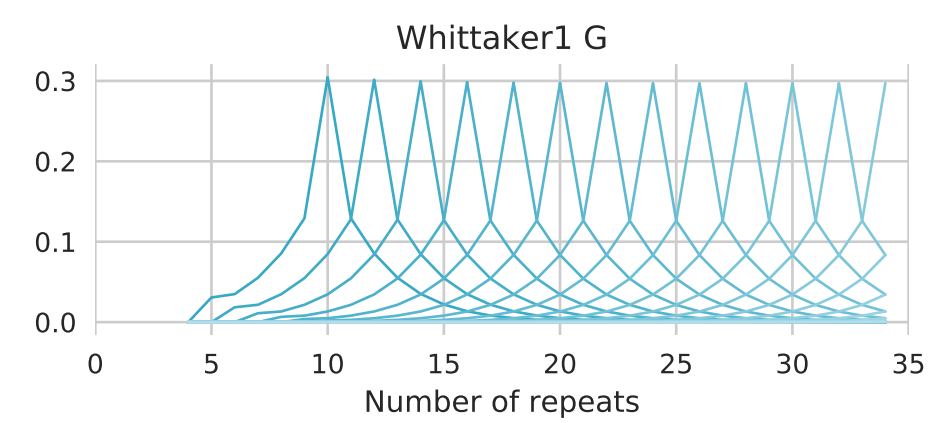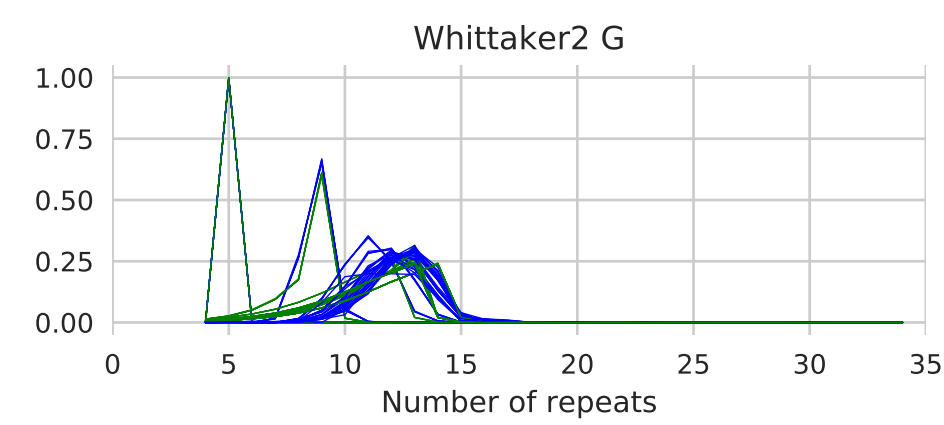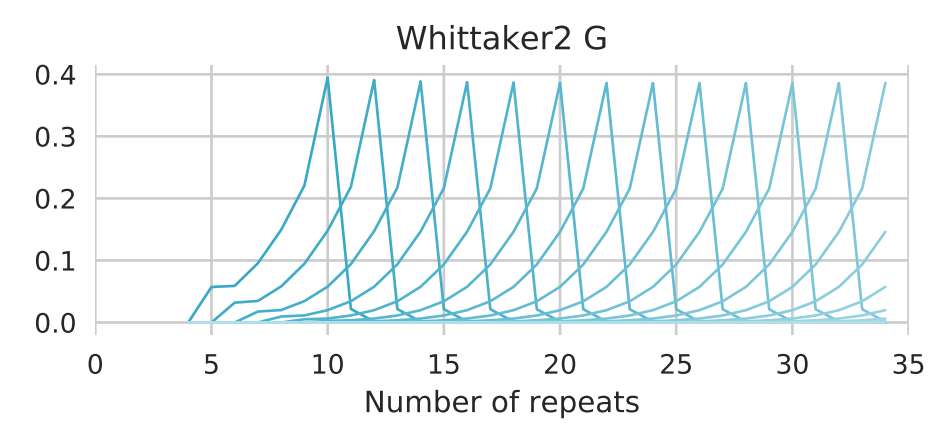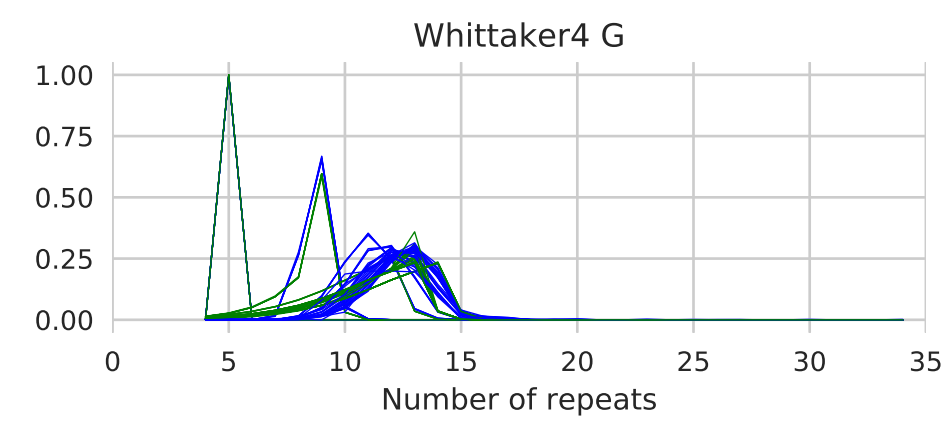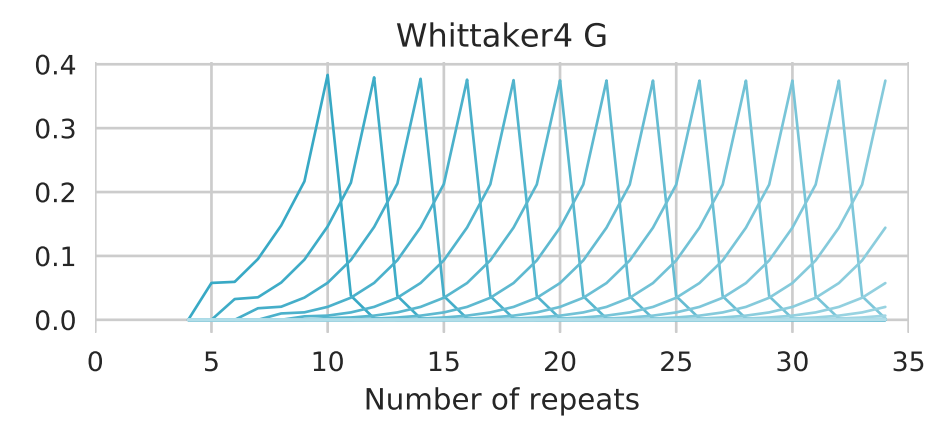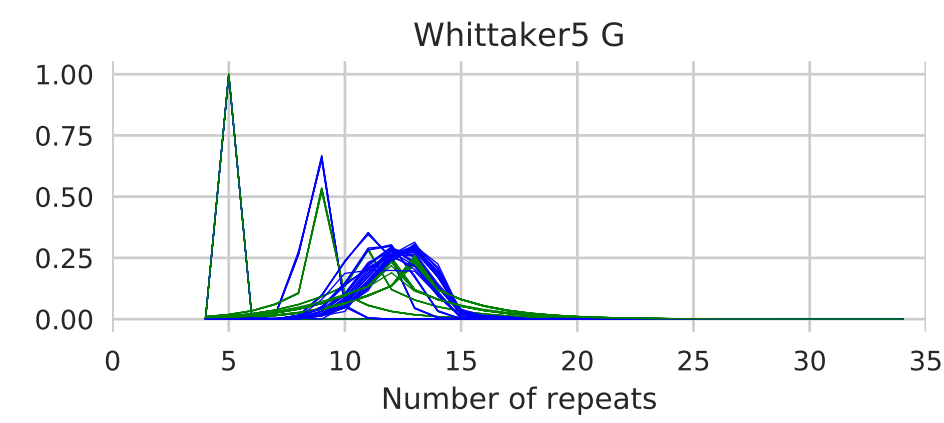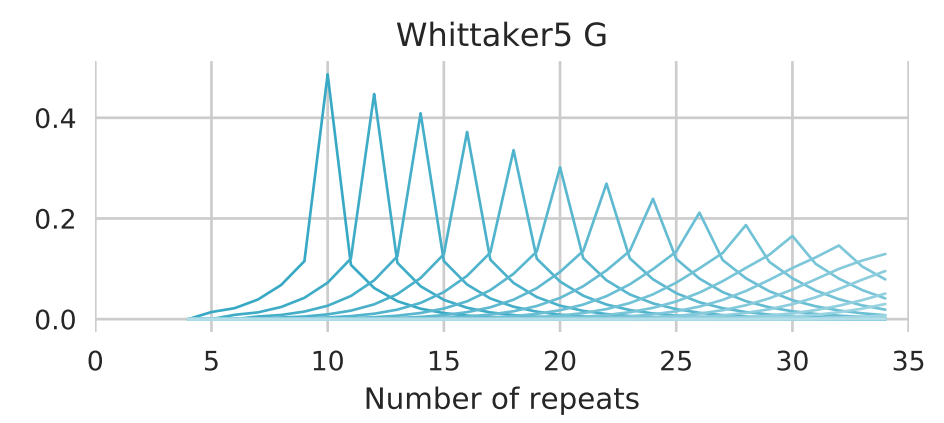

Supplement: Supplementary Data [file gky1318_supplemental_files.zip › Supplemental_Figure_S10_G_series.pdf]

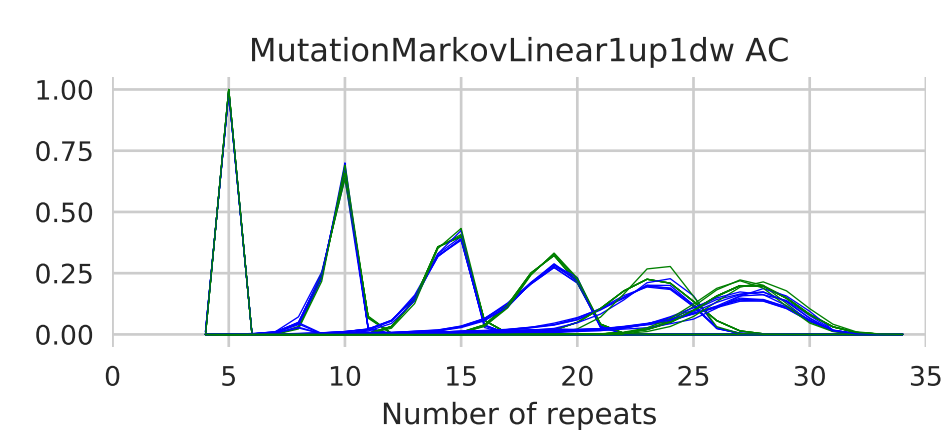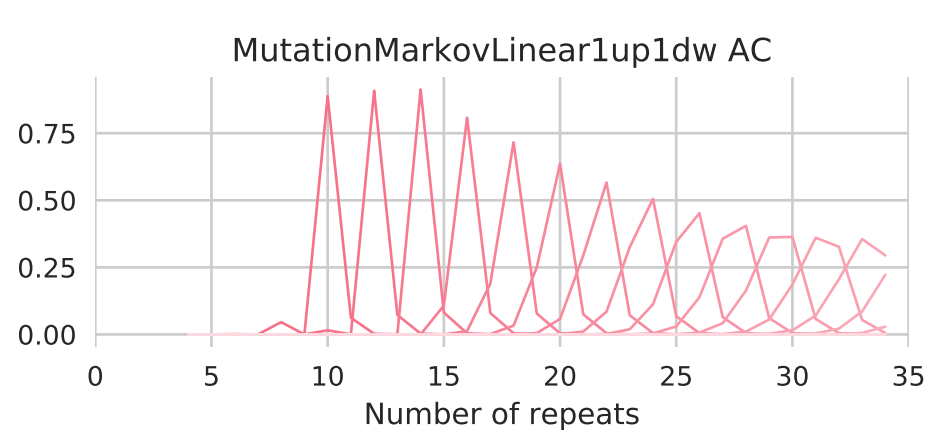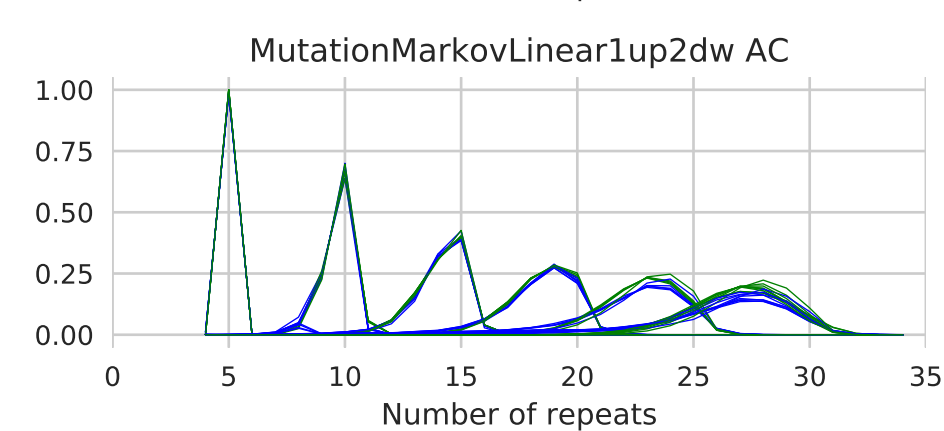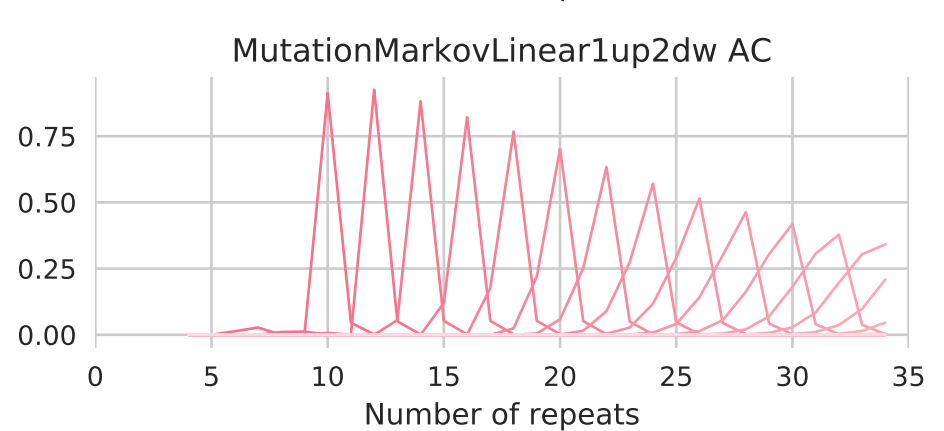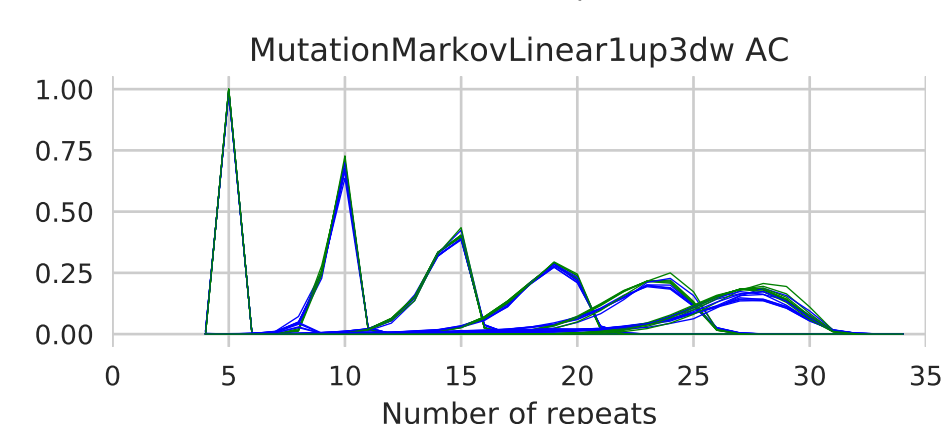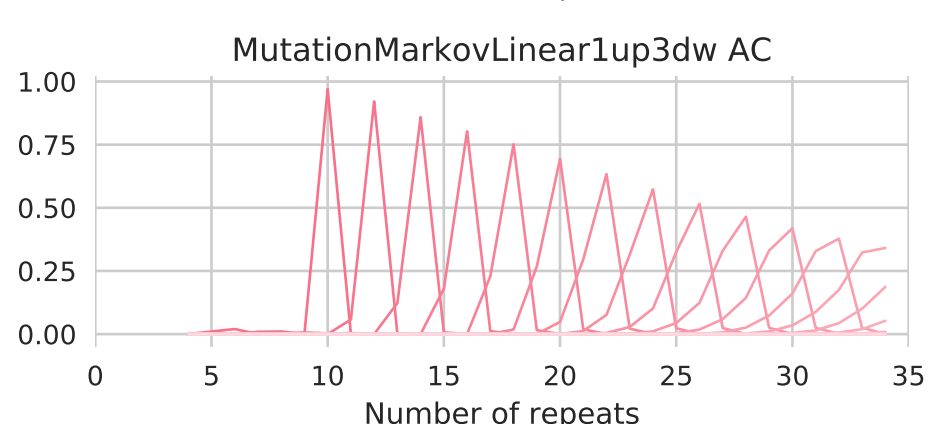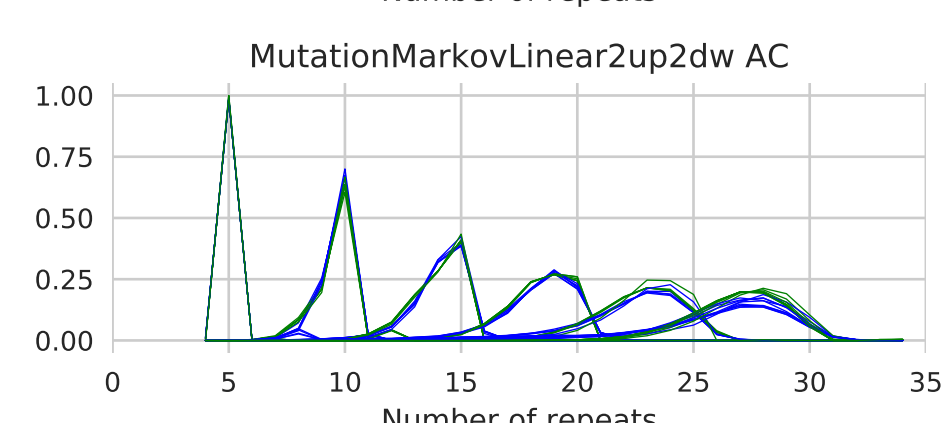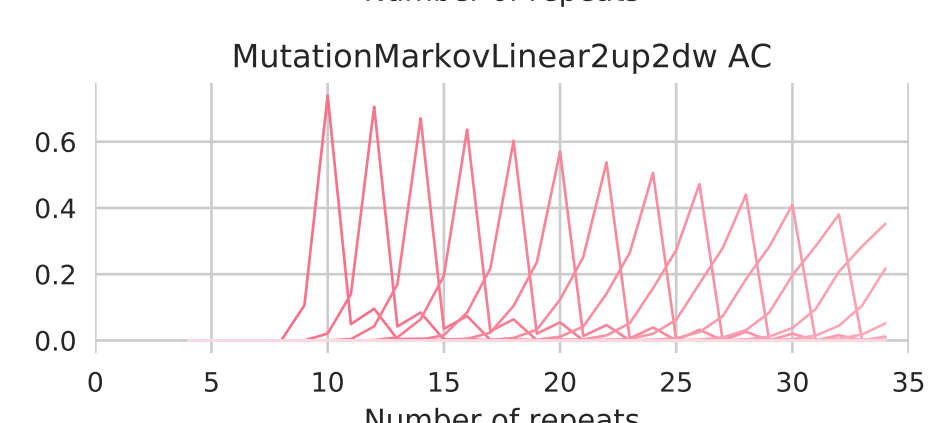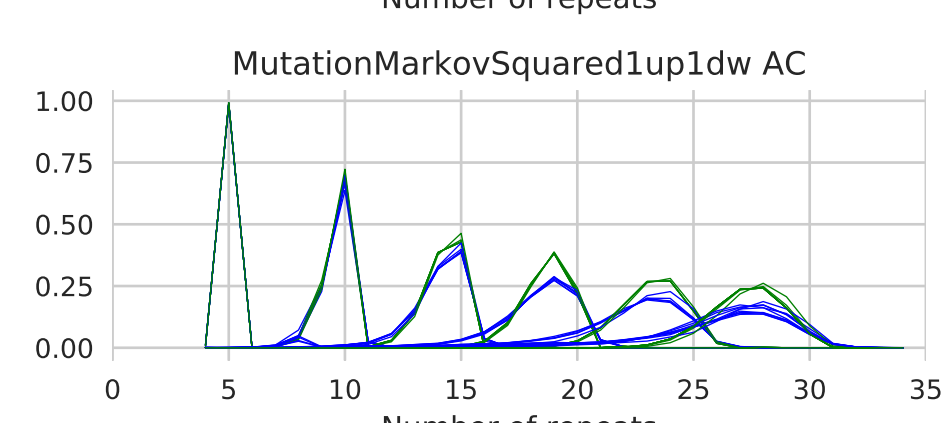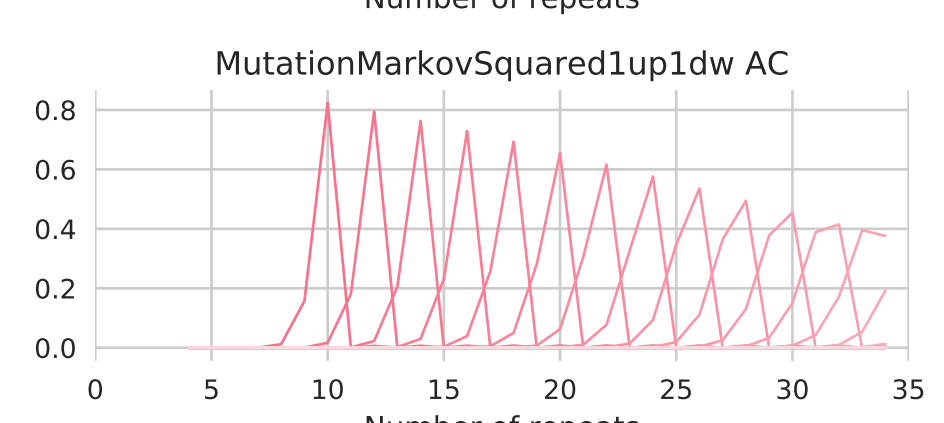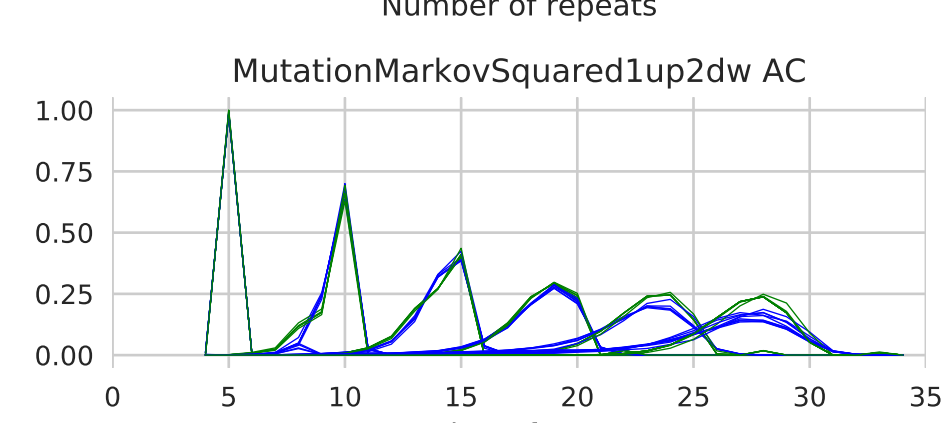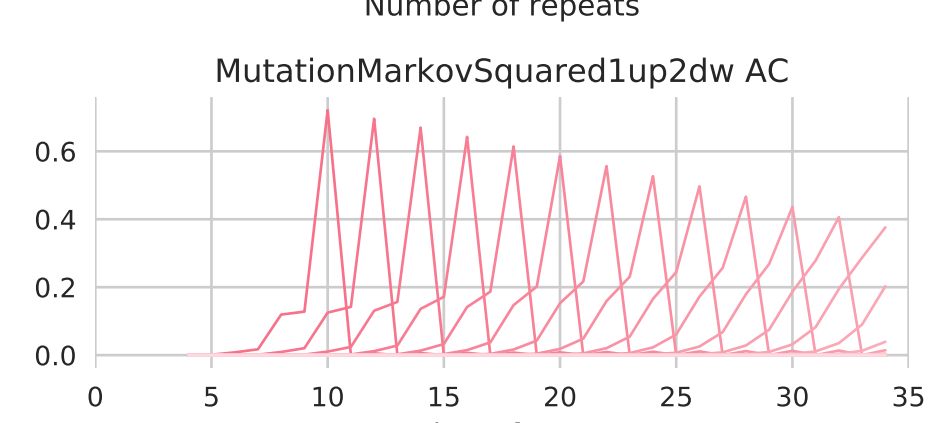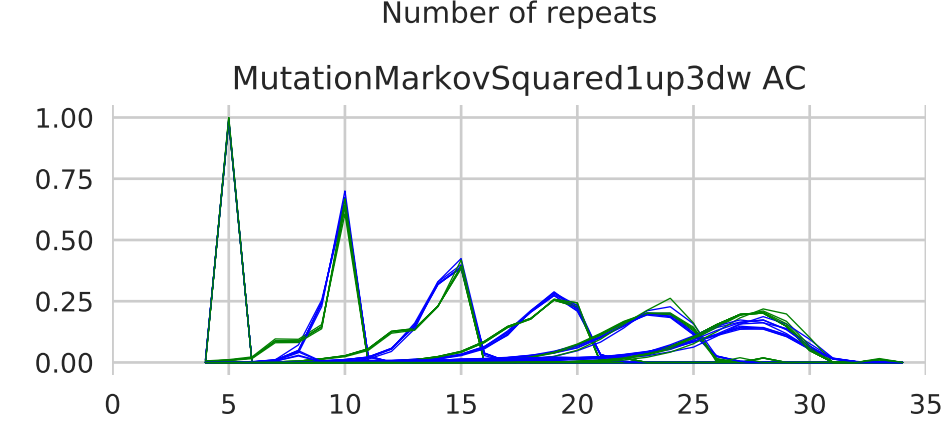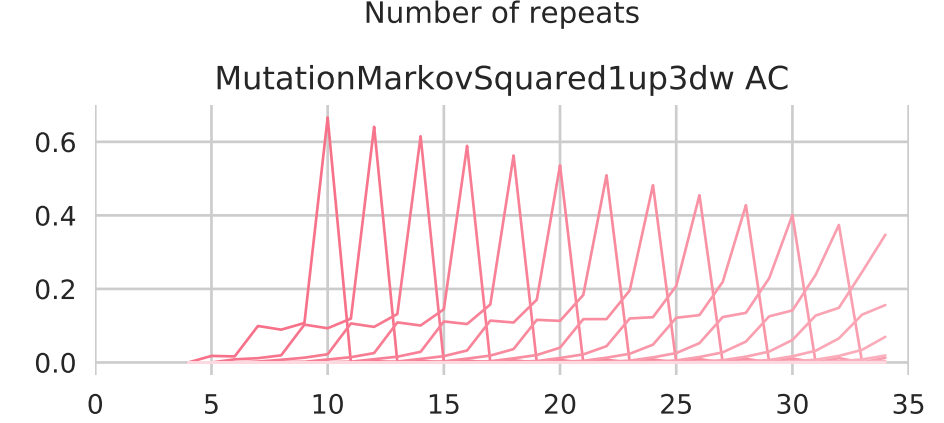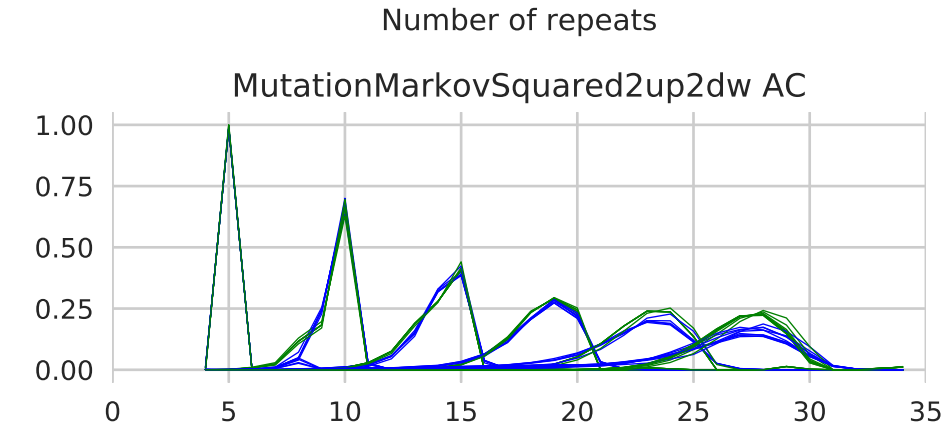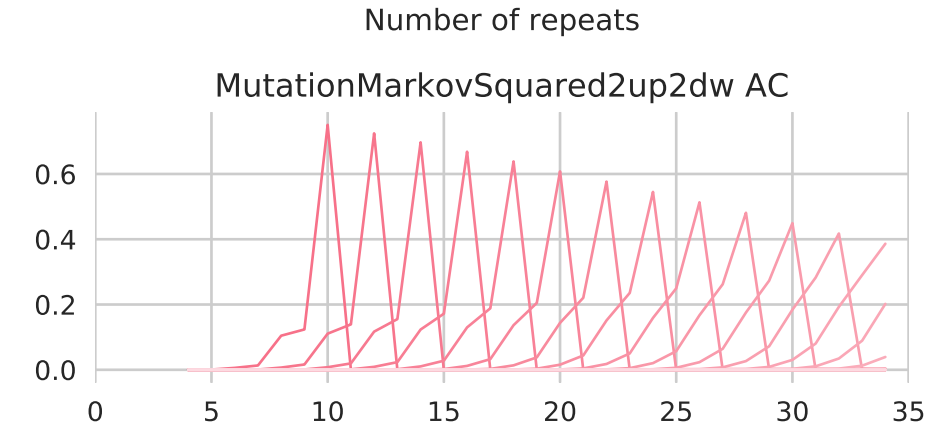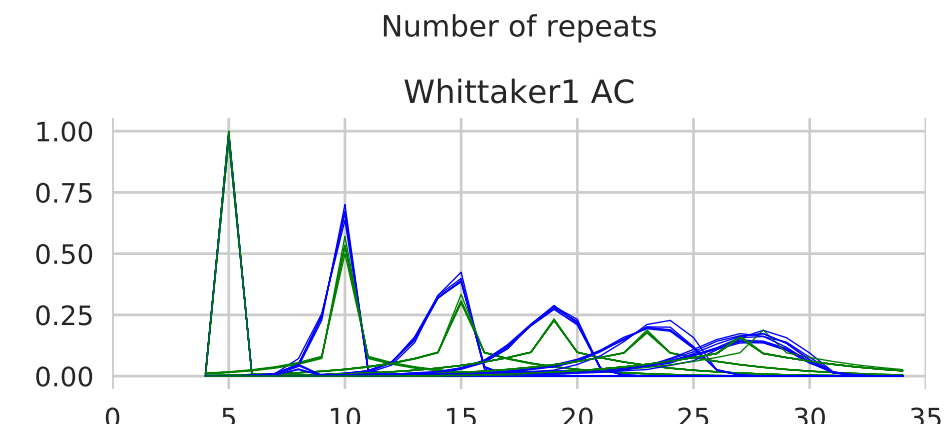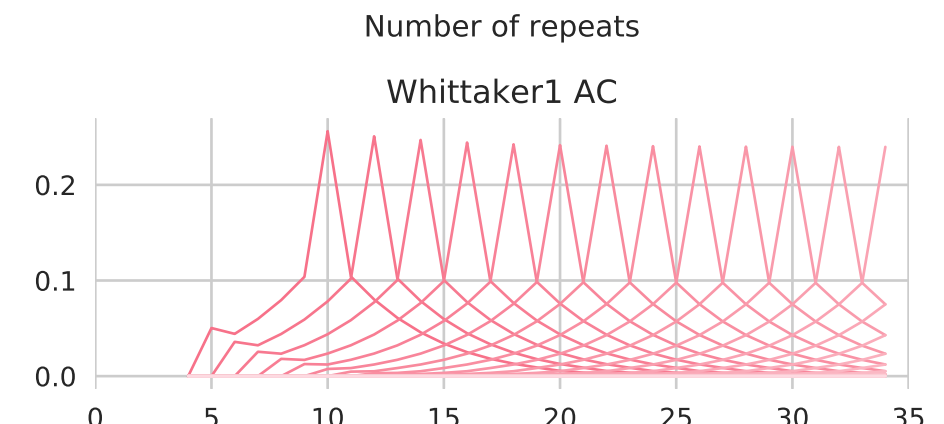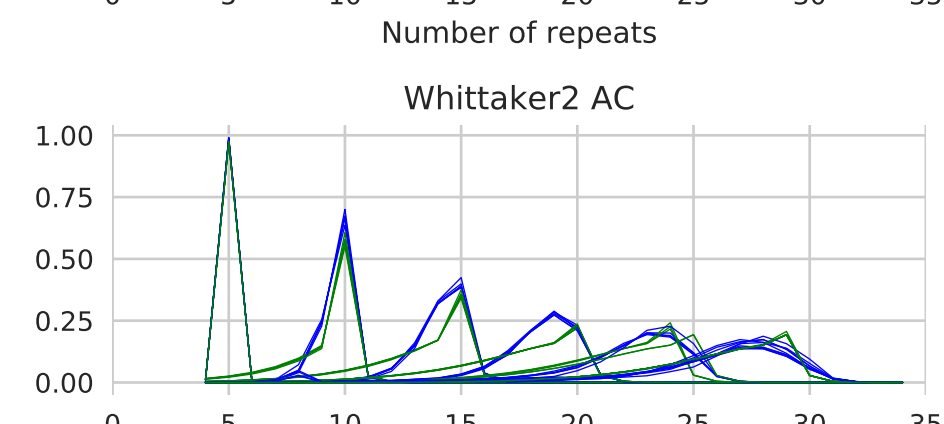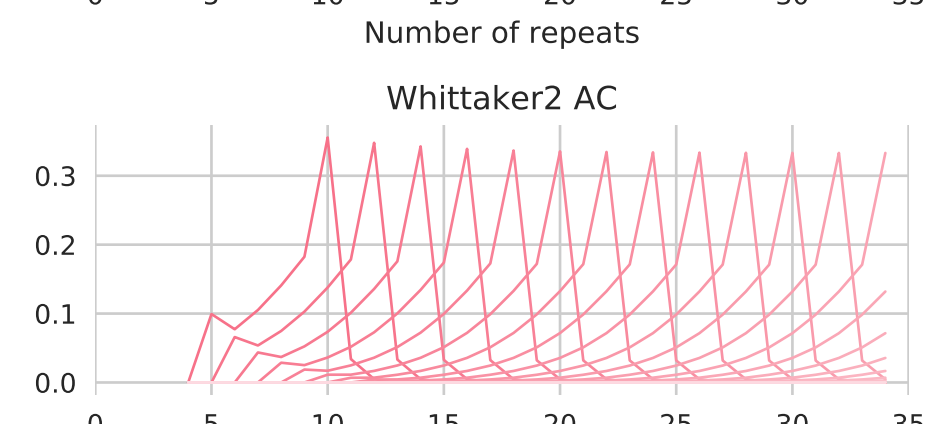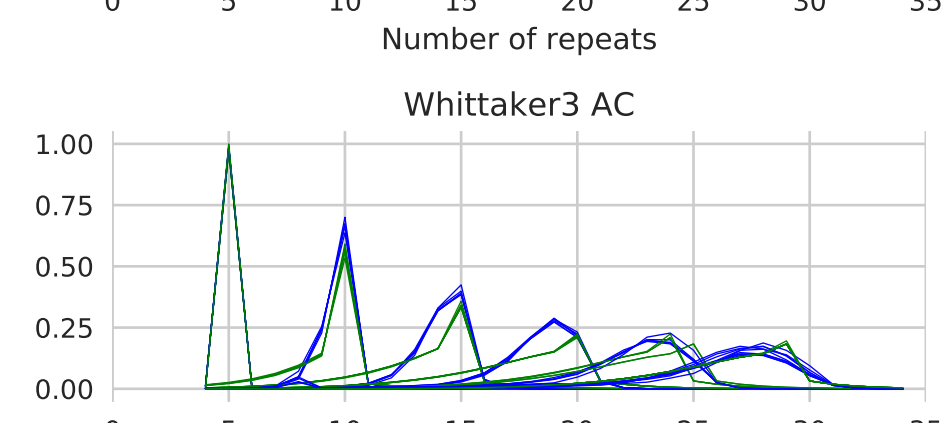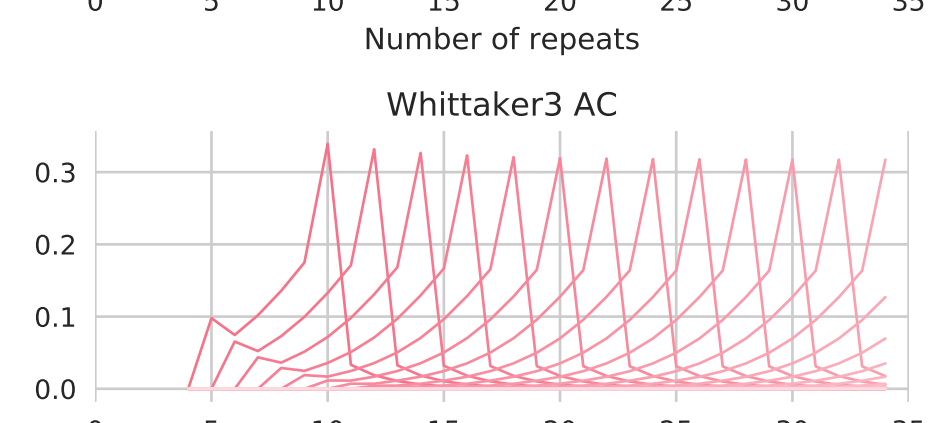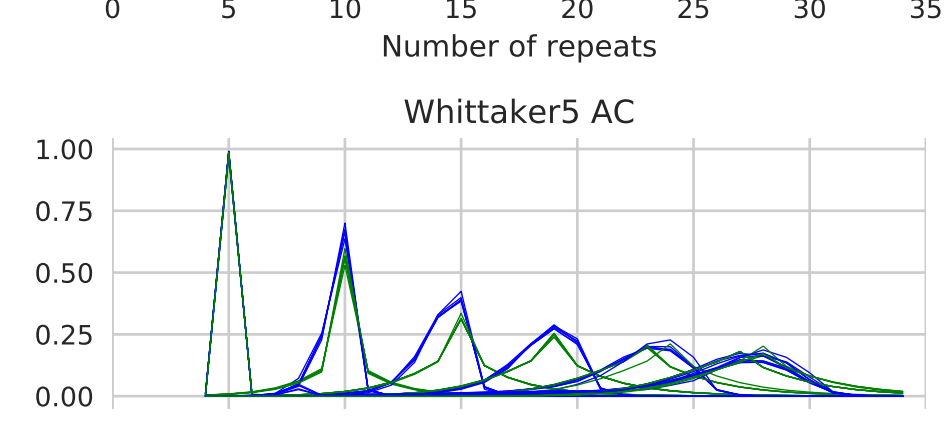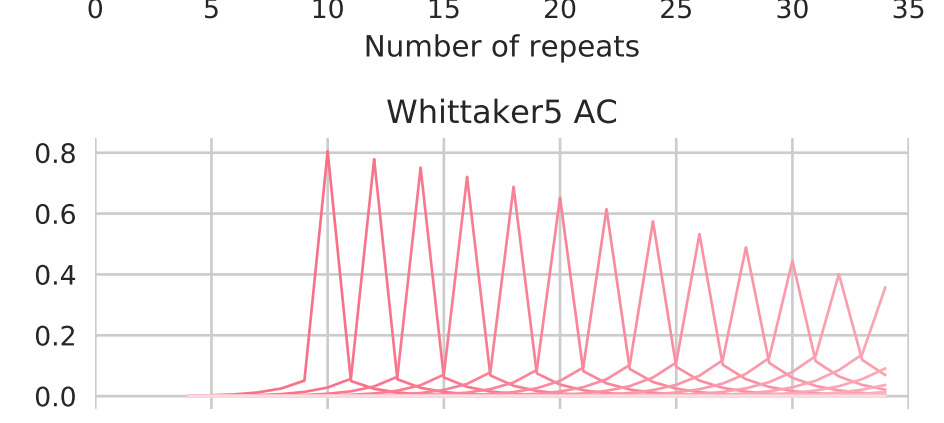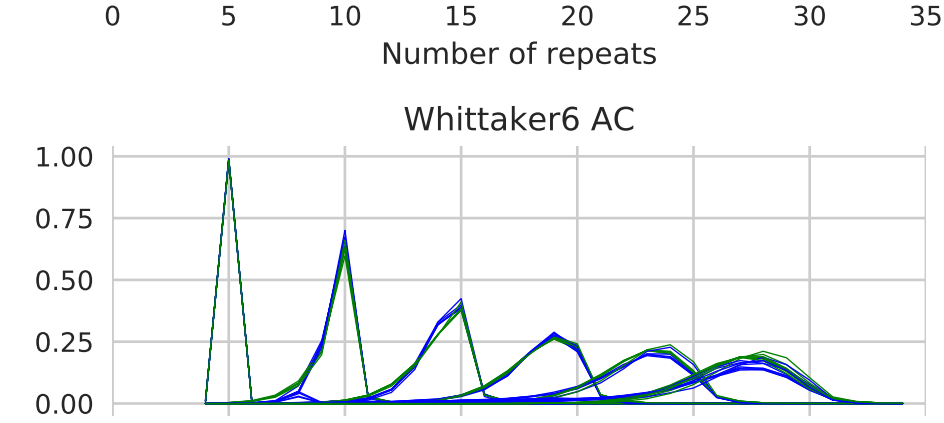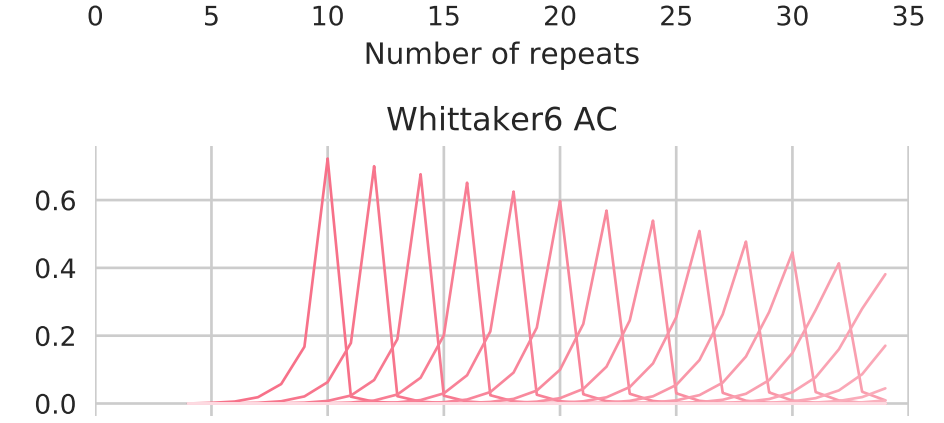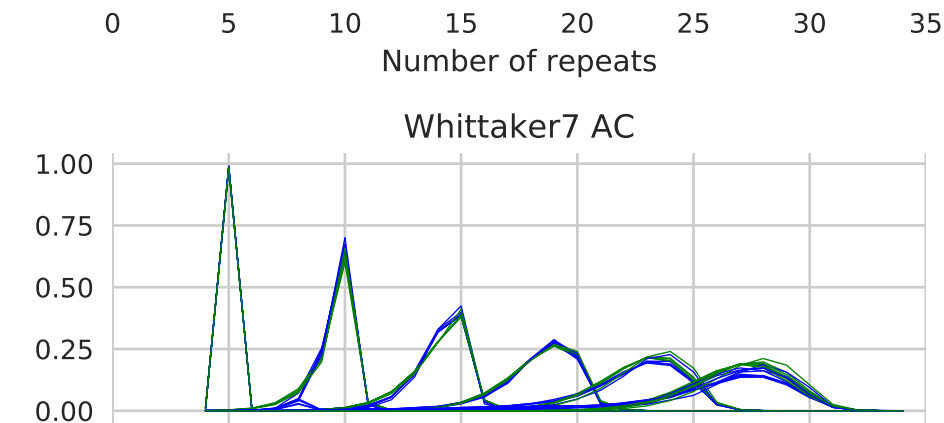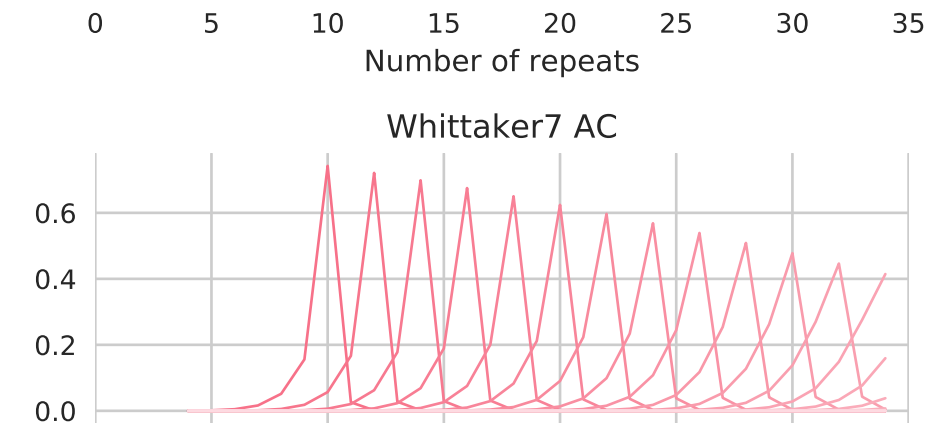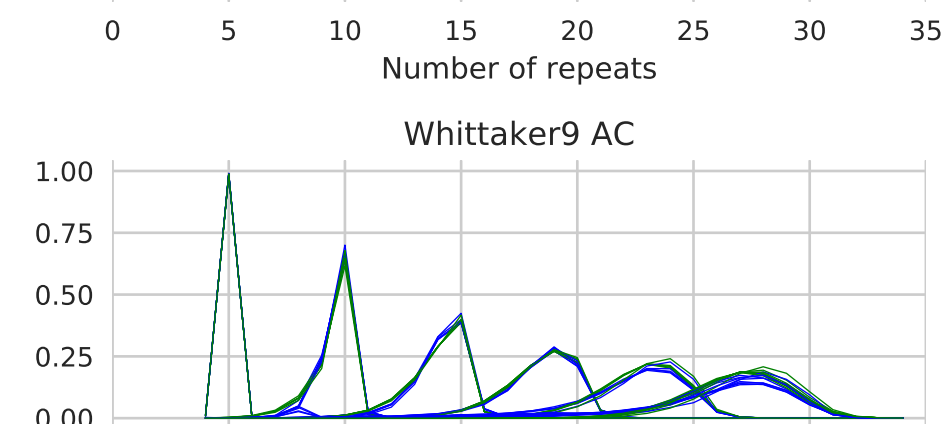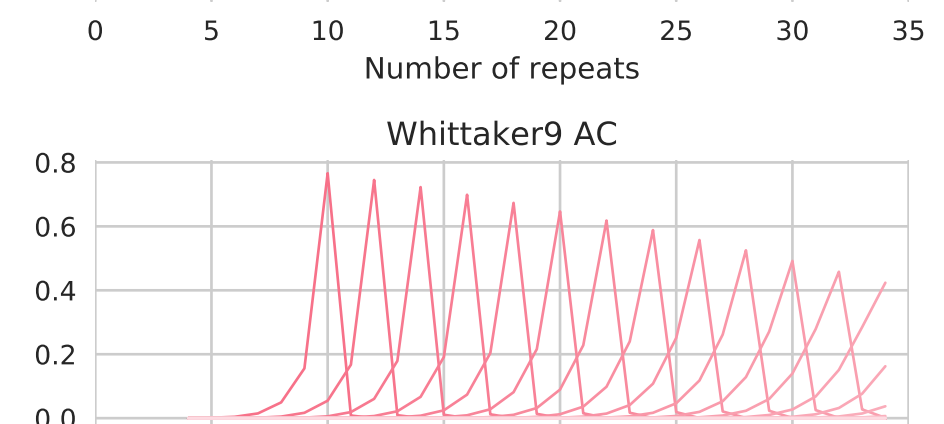

Supplement: Supplementary Data [file gky1318_supplemental_files.zip › Supplemental_Figure_S6_AC_series.pdf]

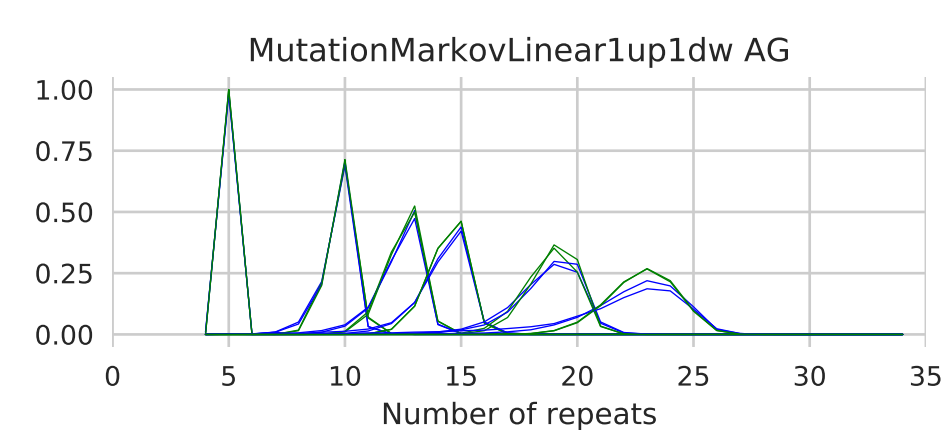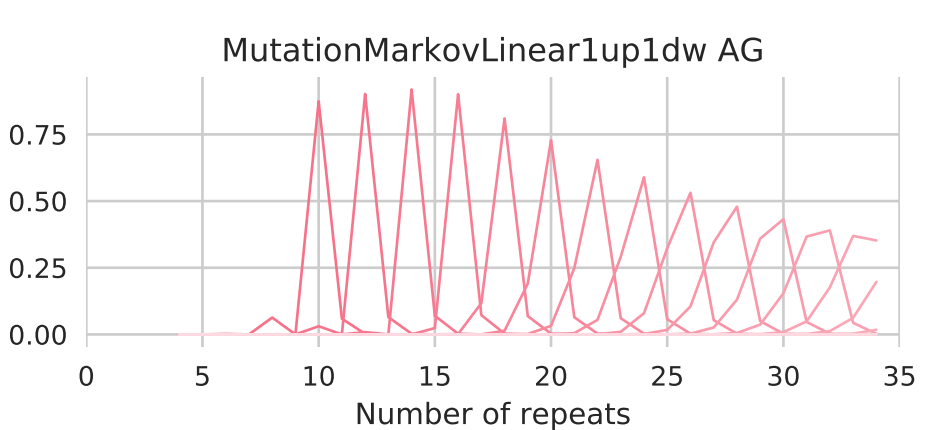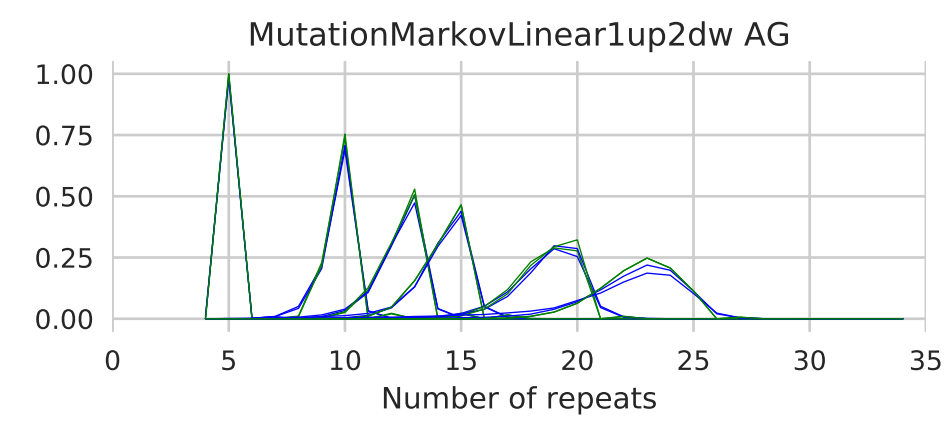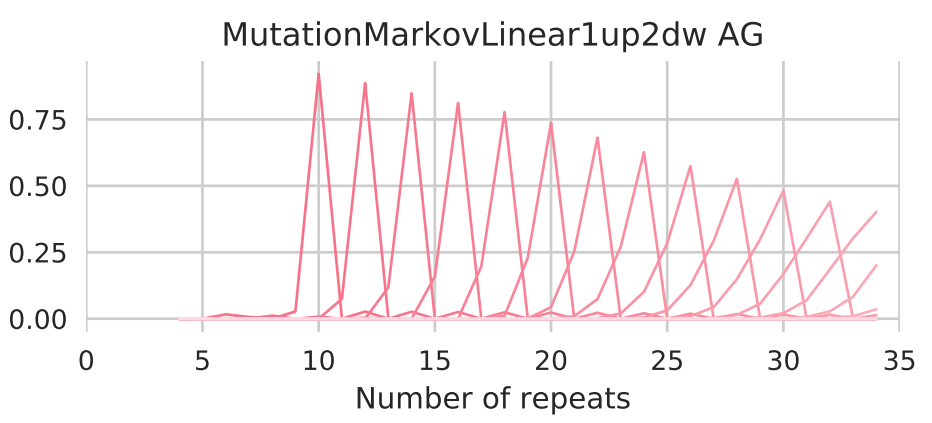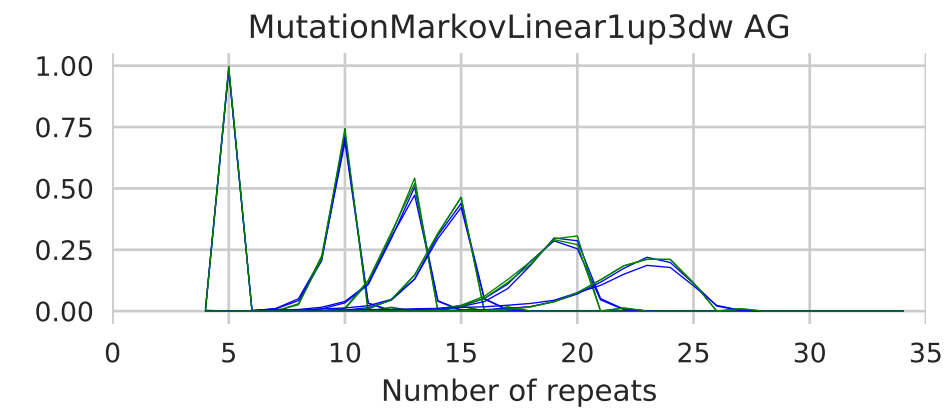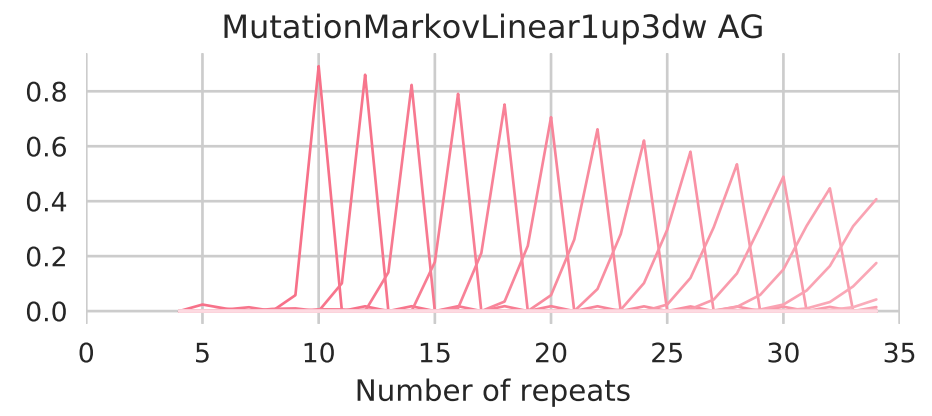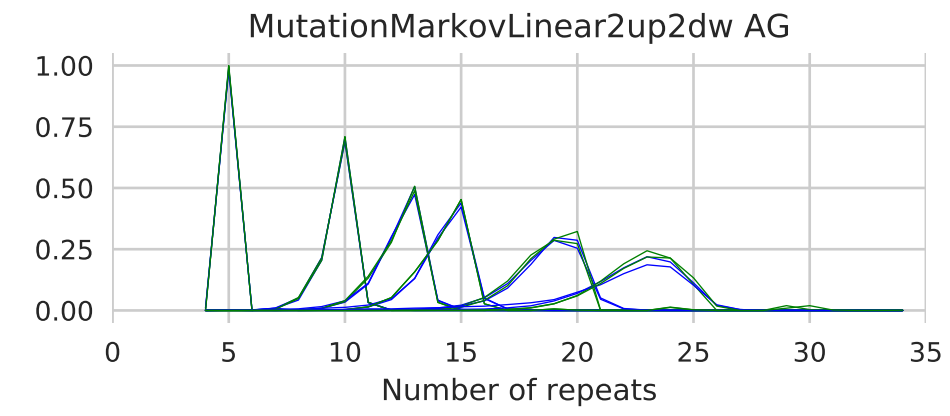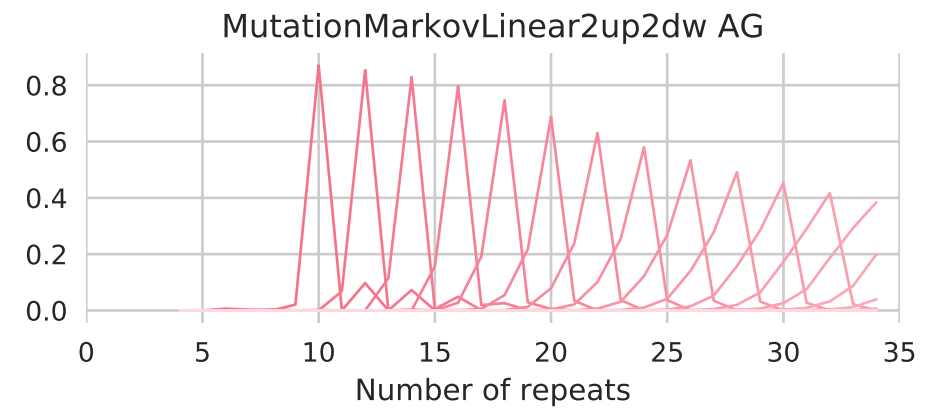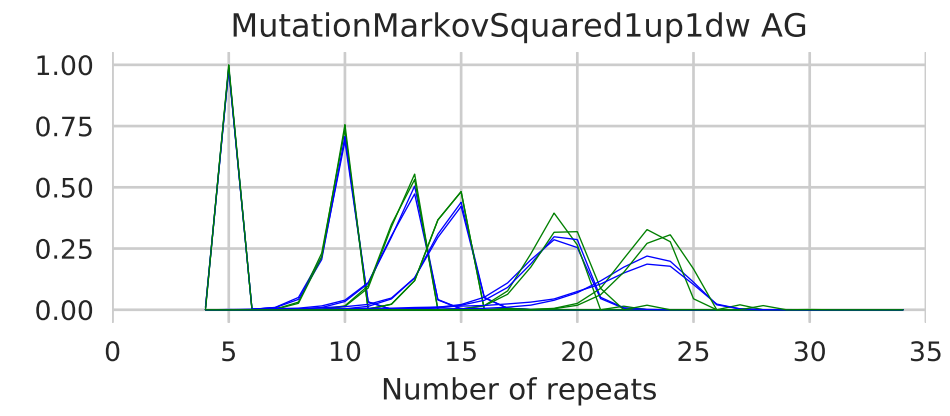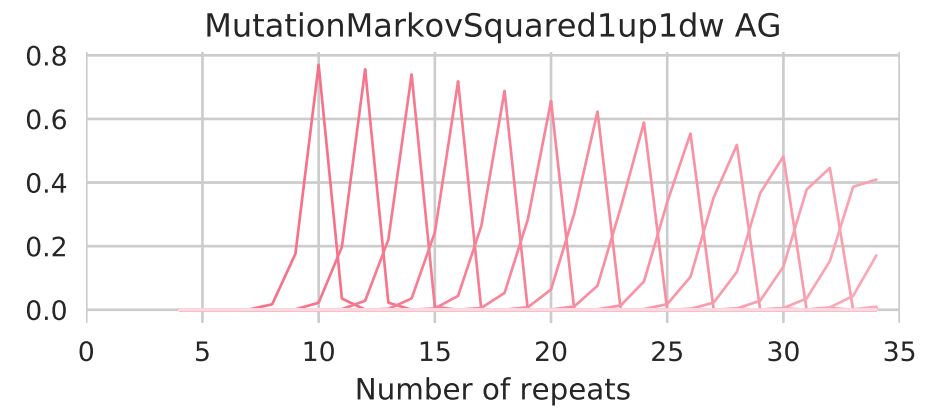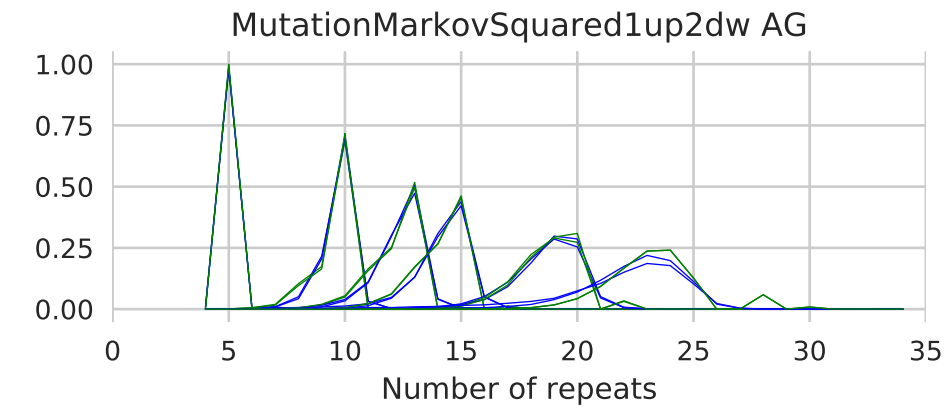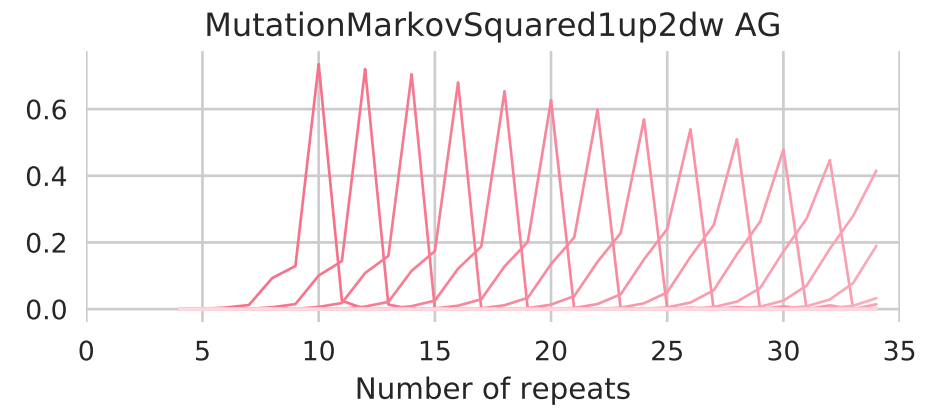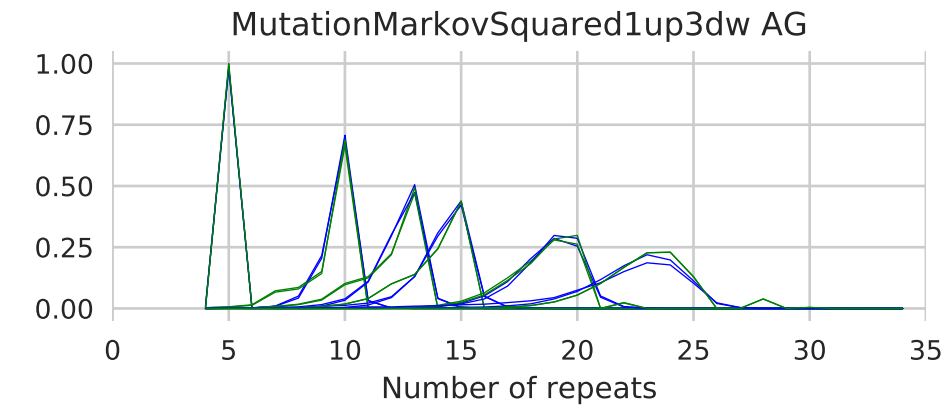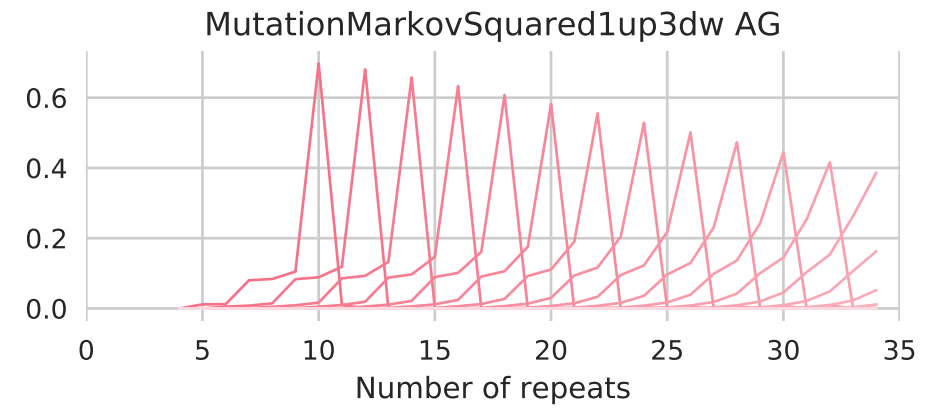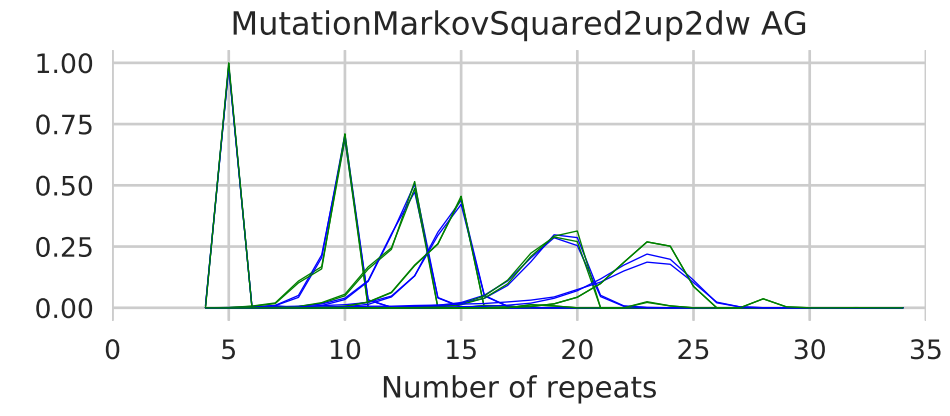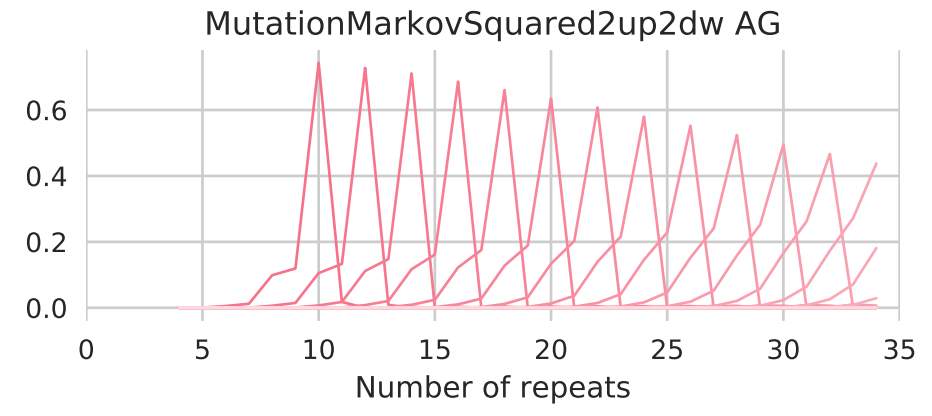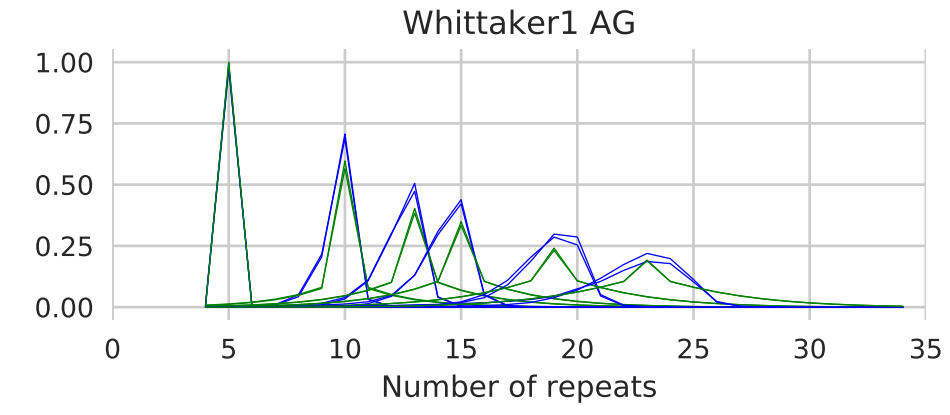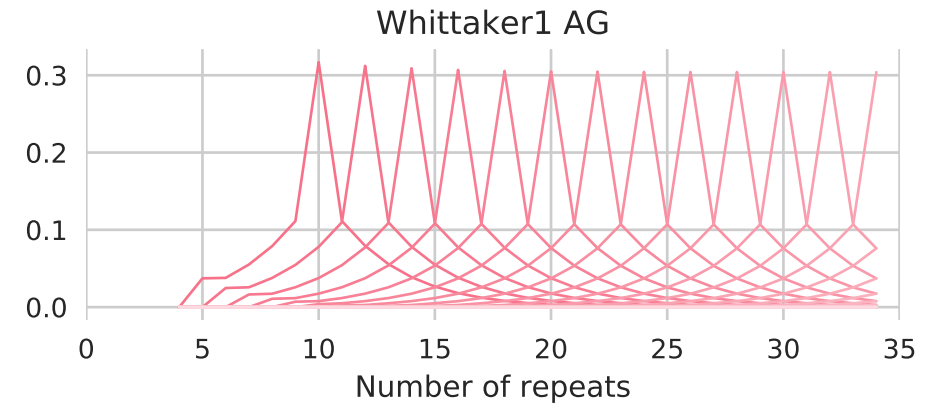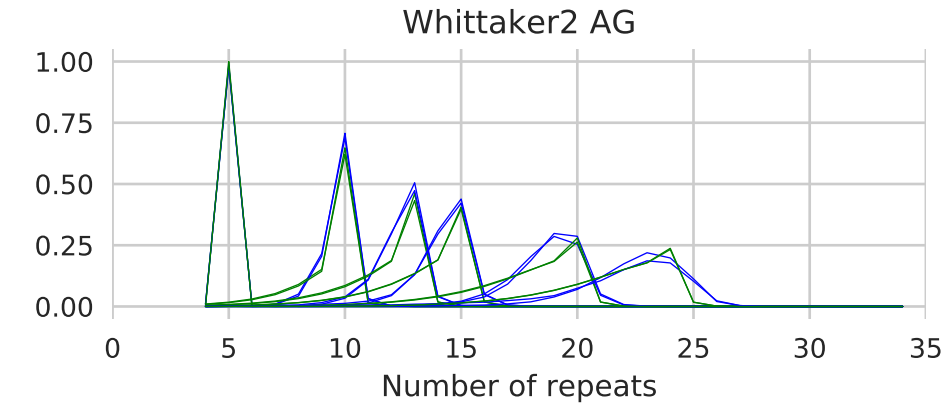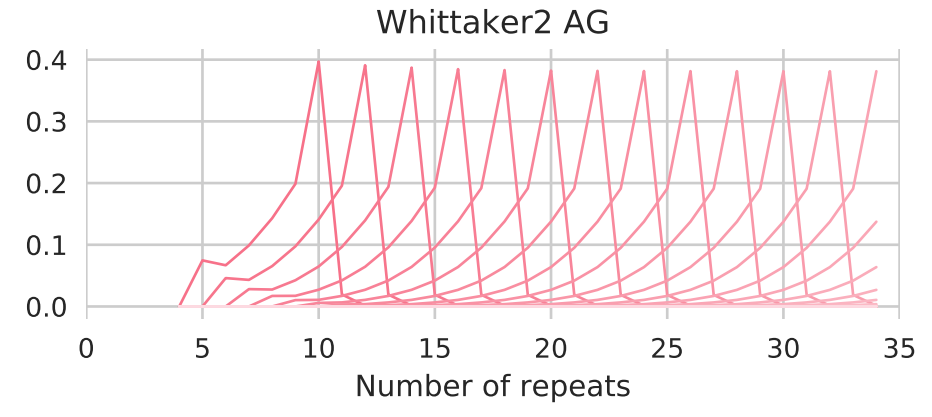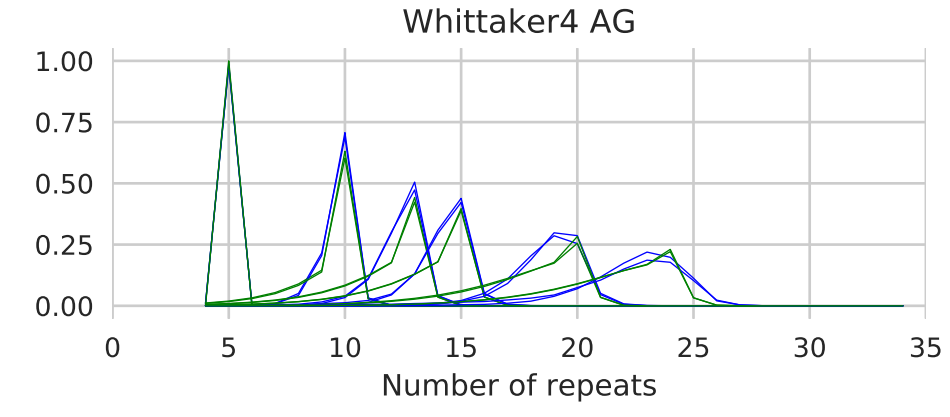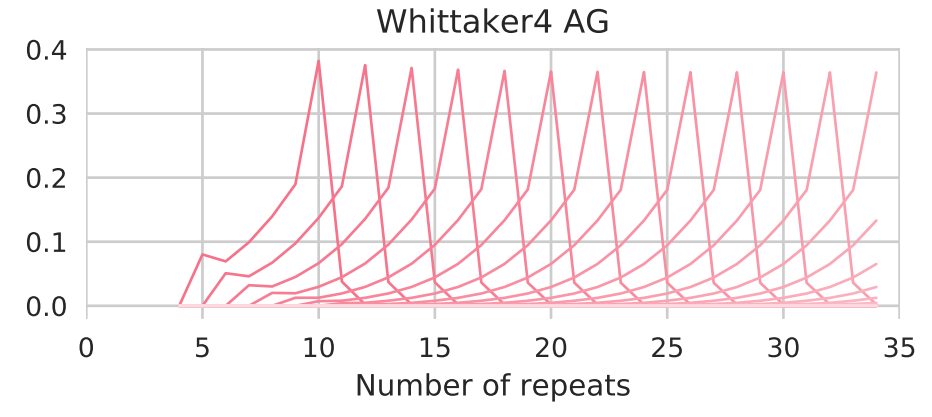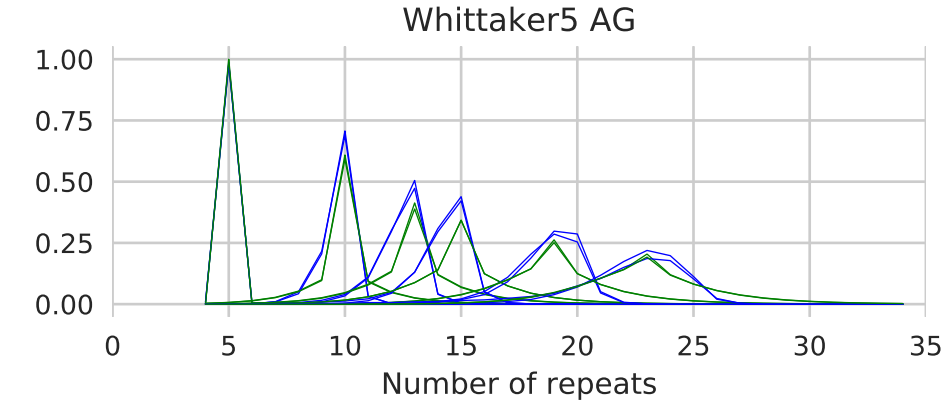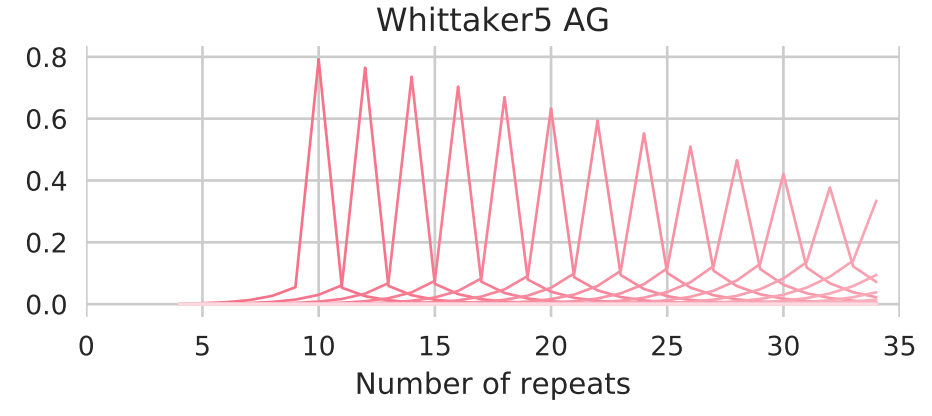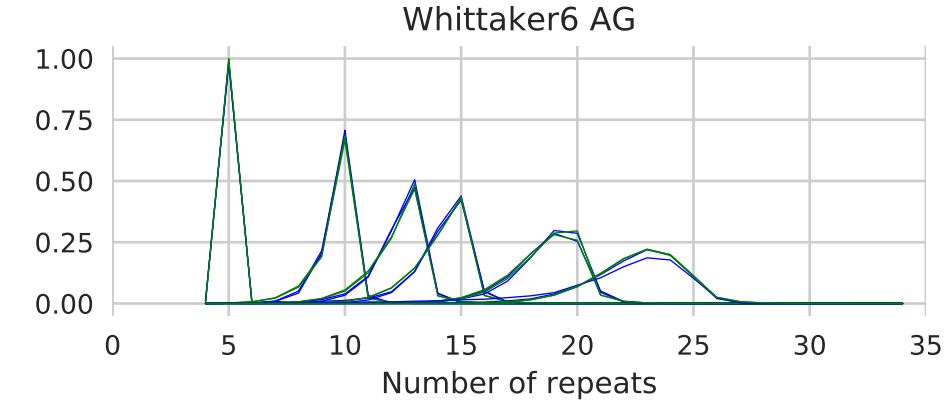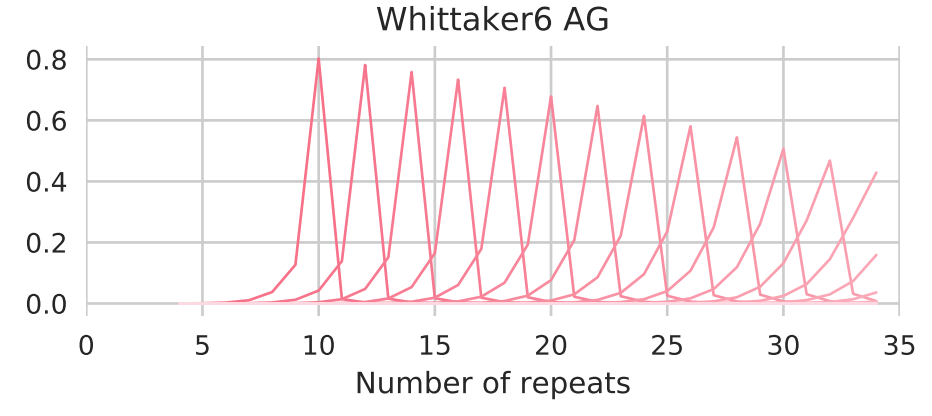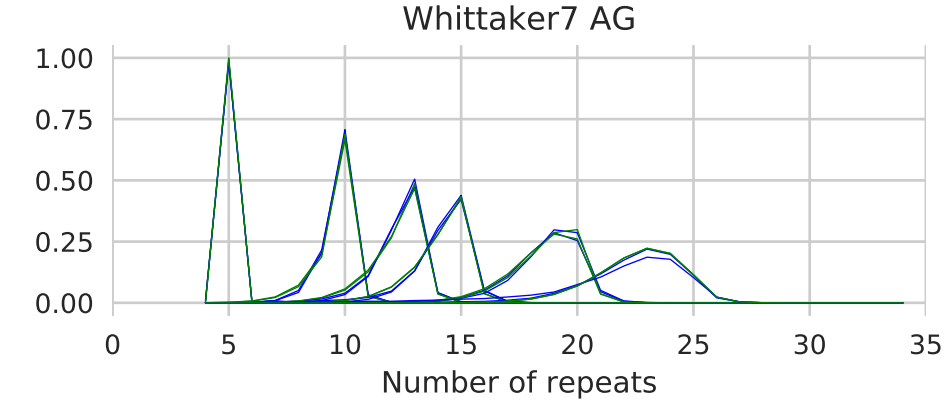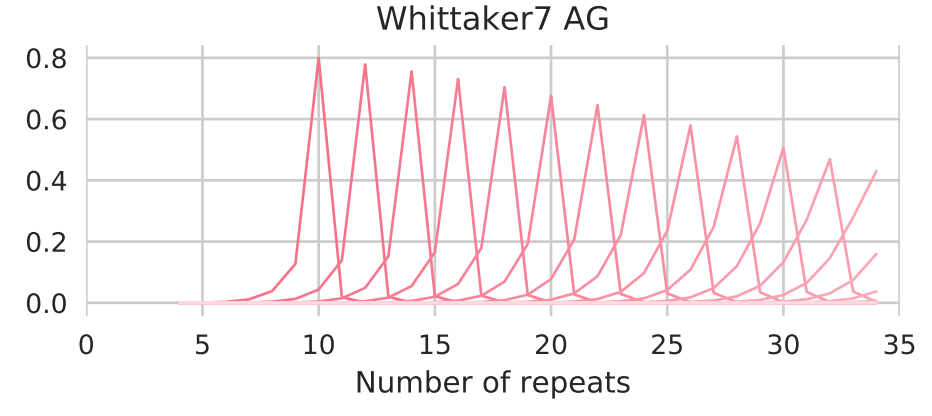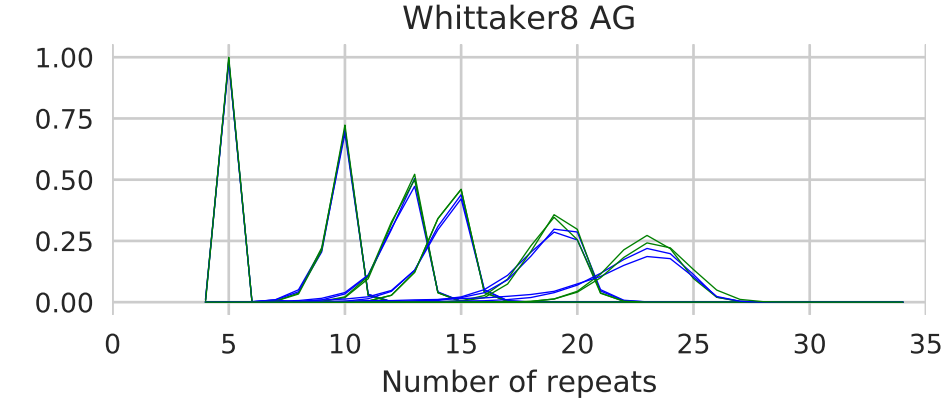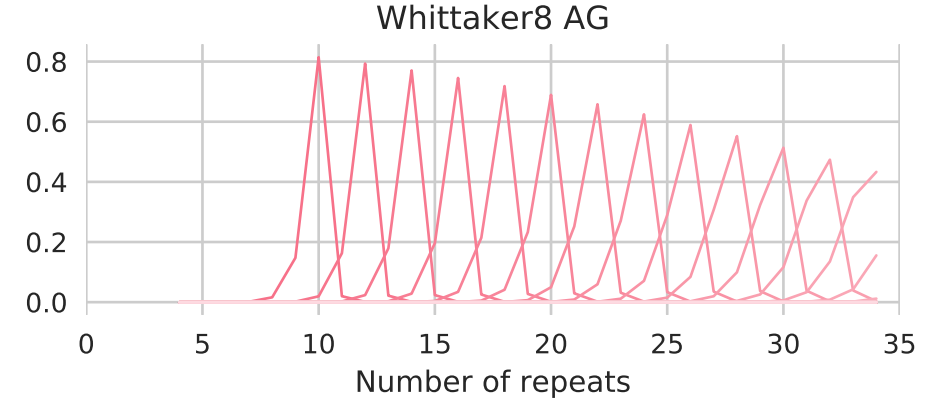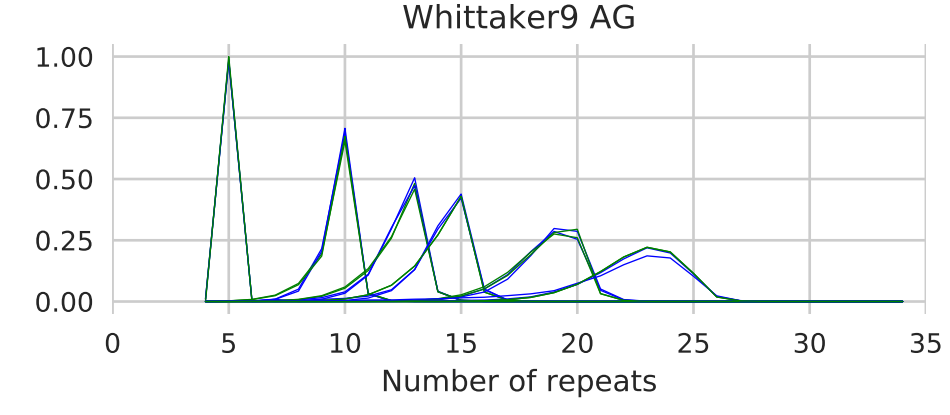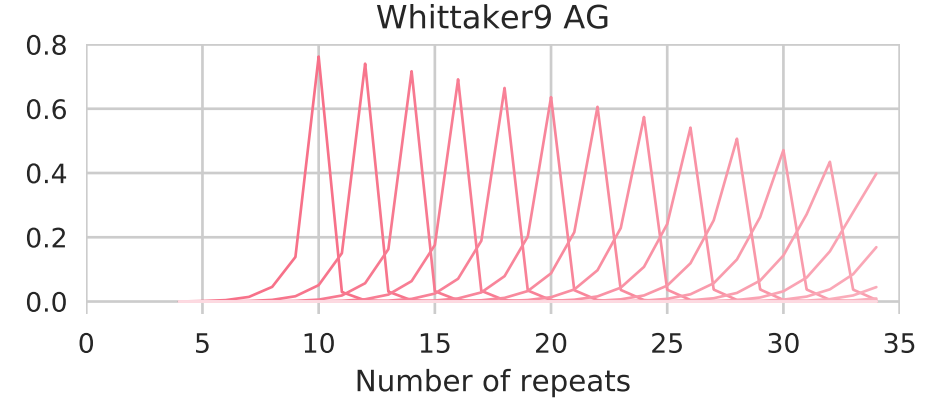

Supplement: Supplementary Data [file gky1318_supplemental_files.zip › Supplemental_Figure_S7_AG_series.pdf]

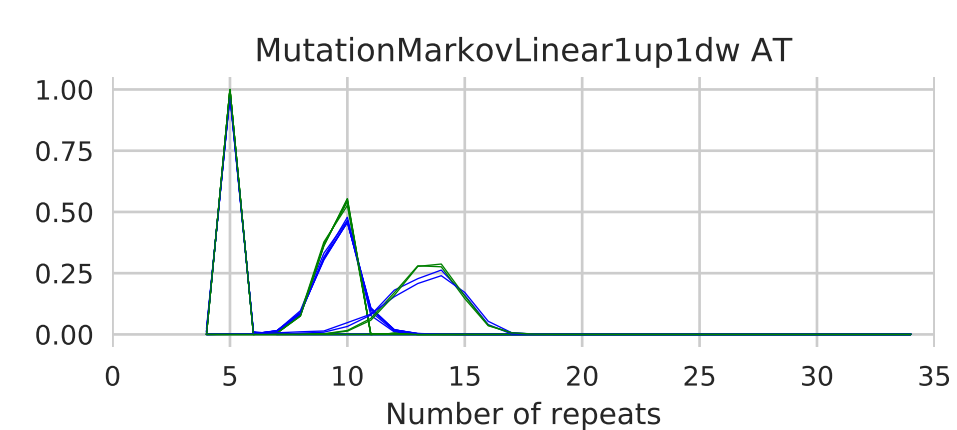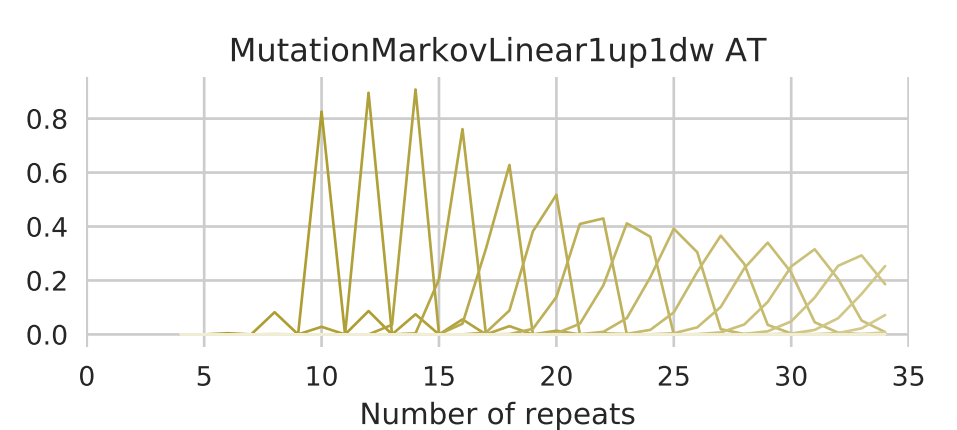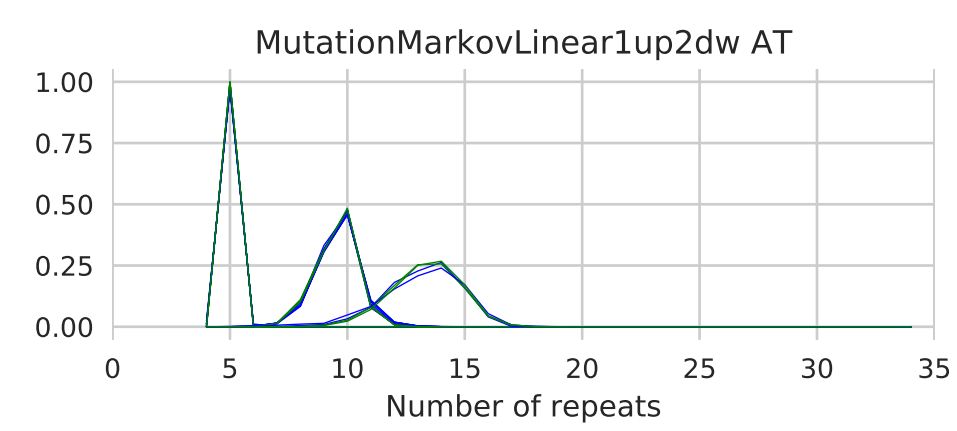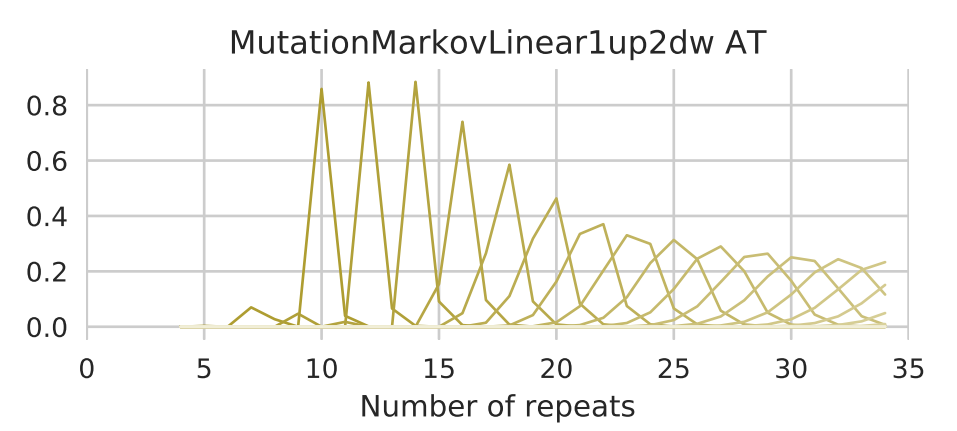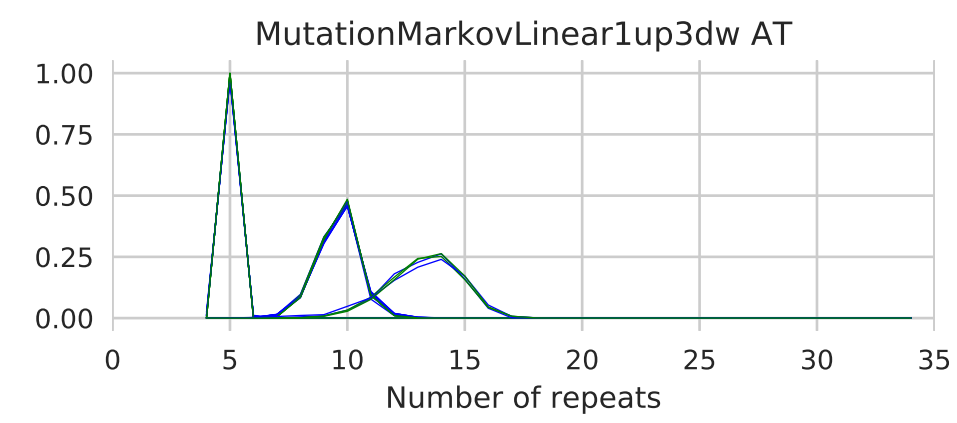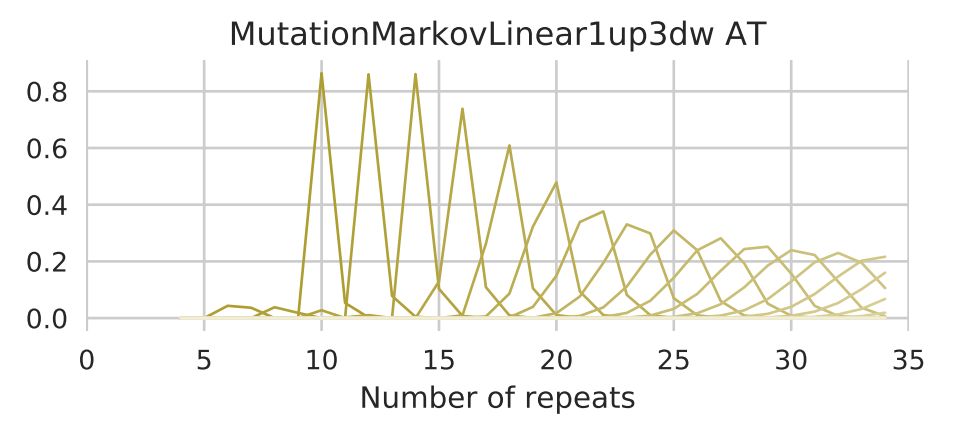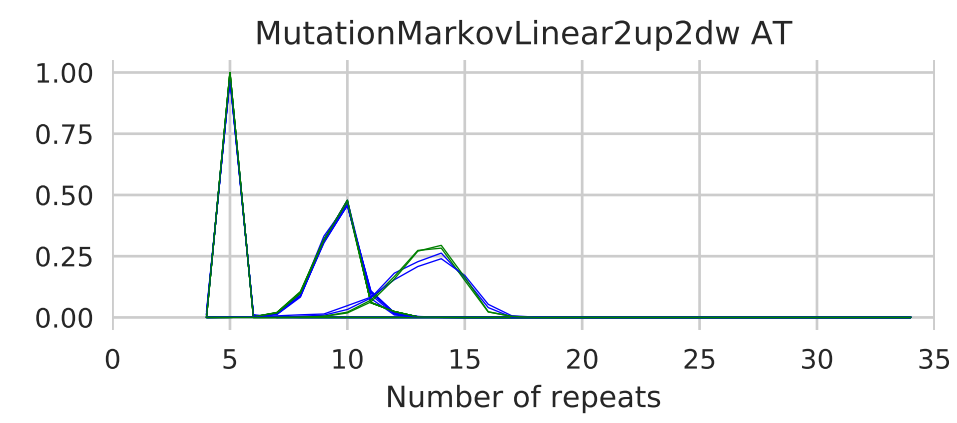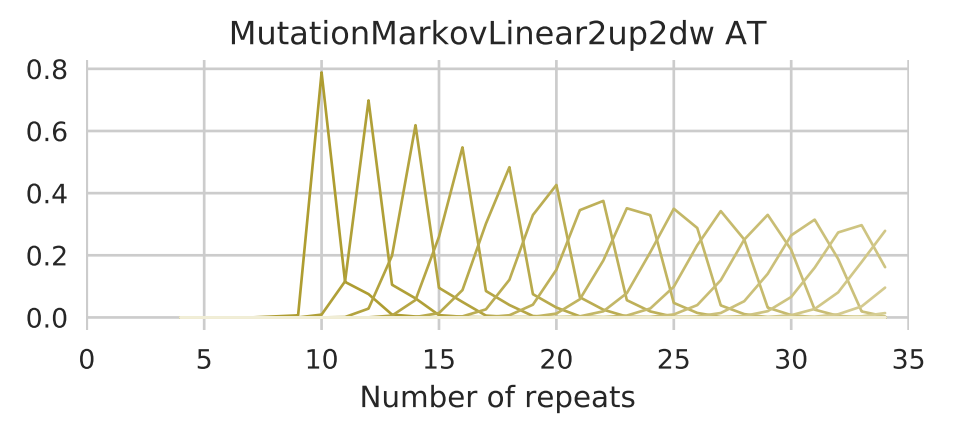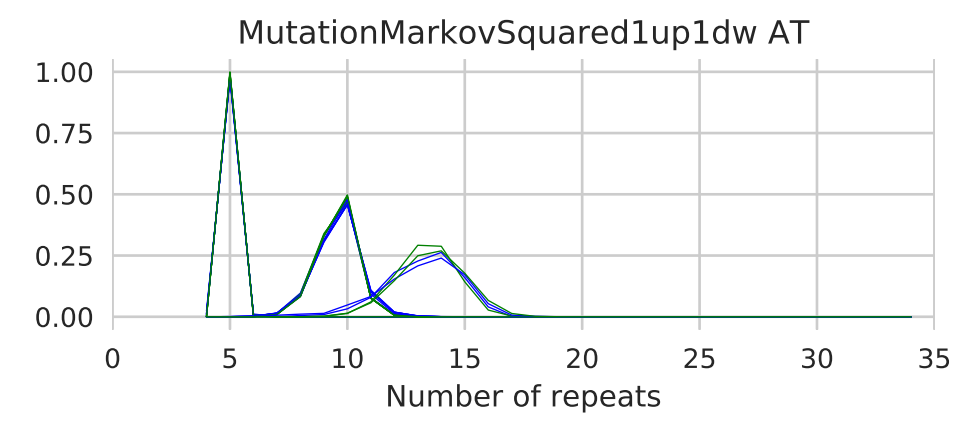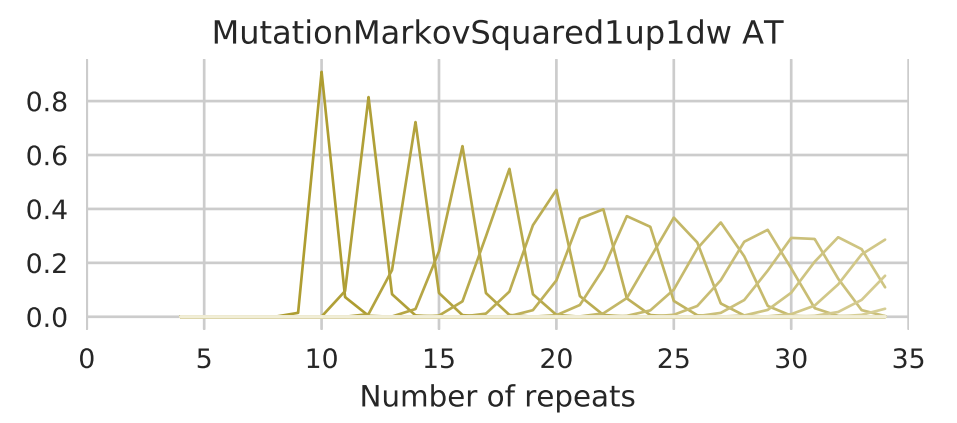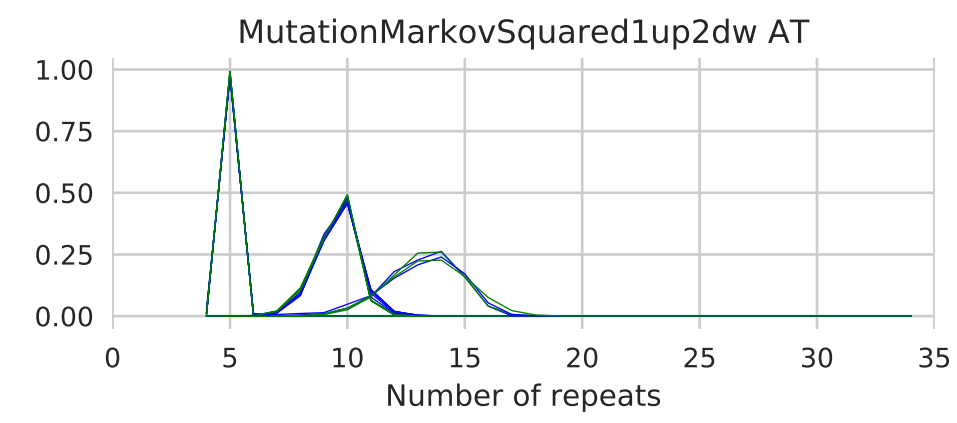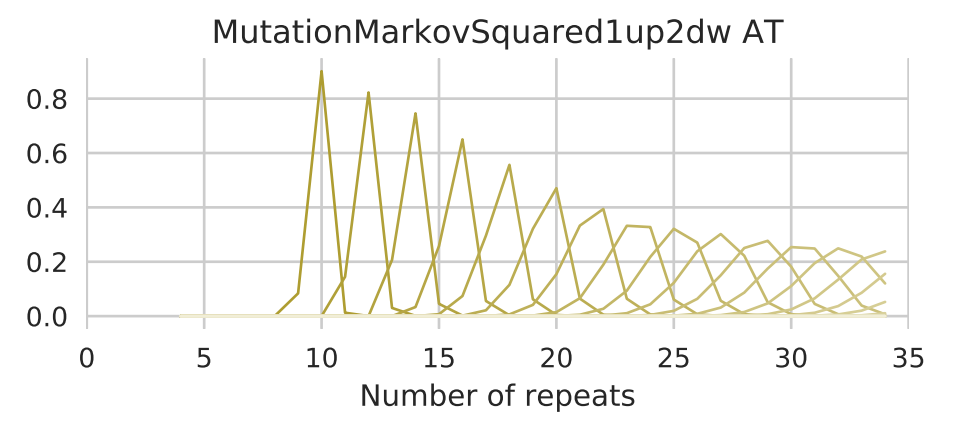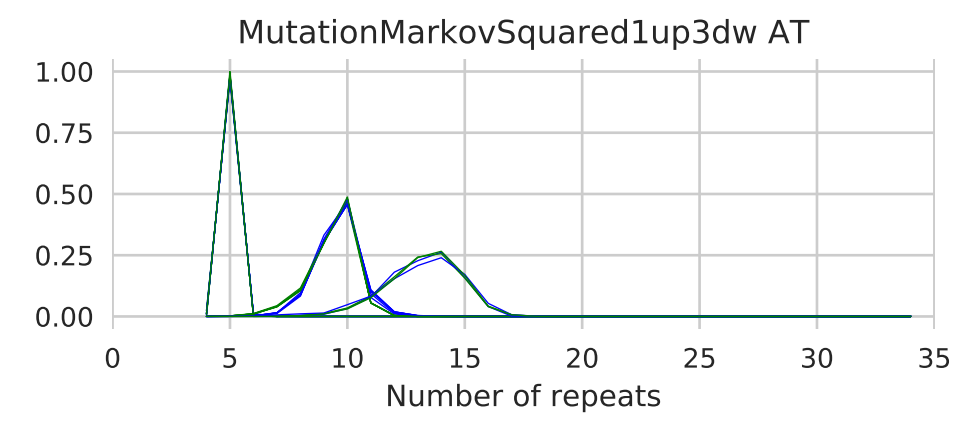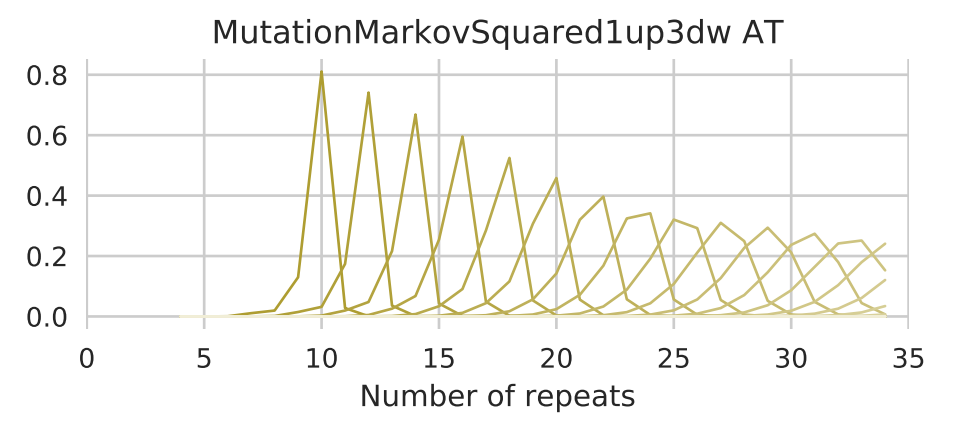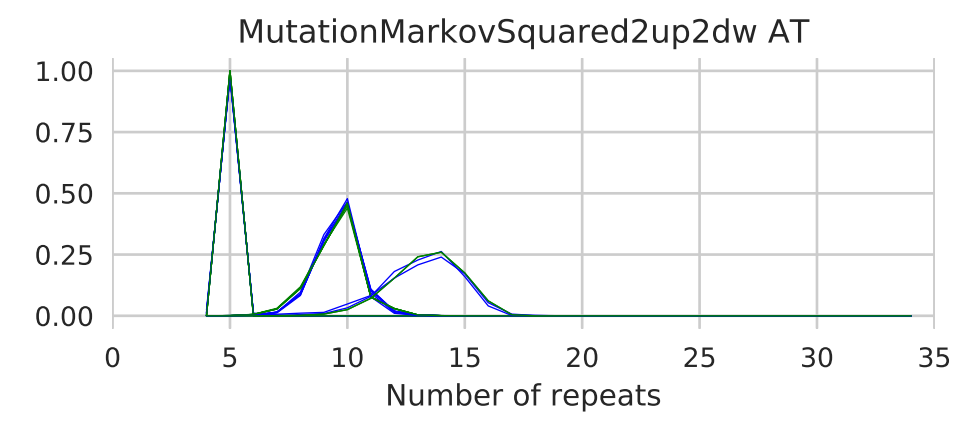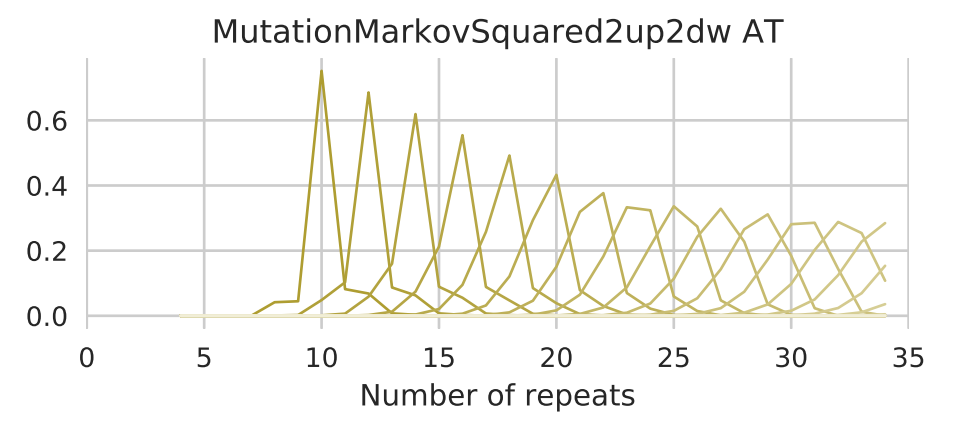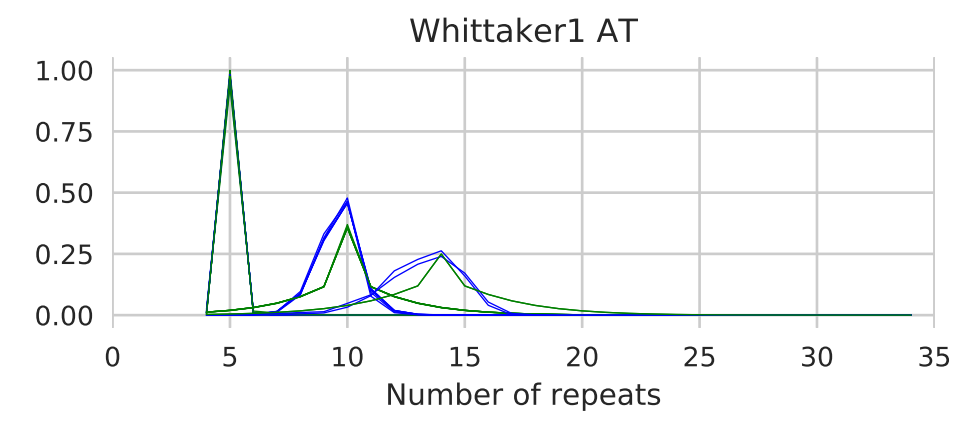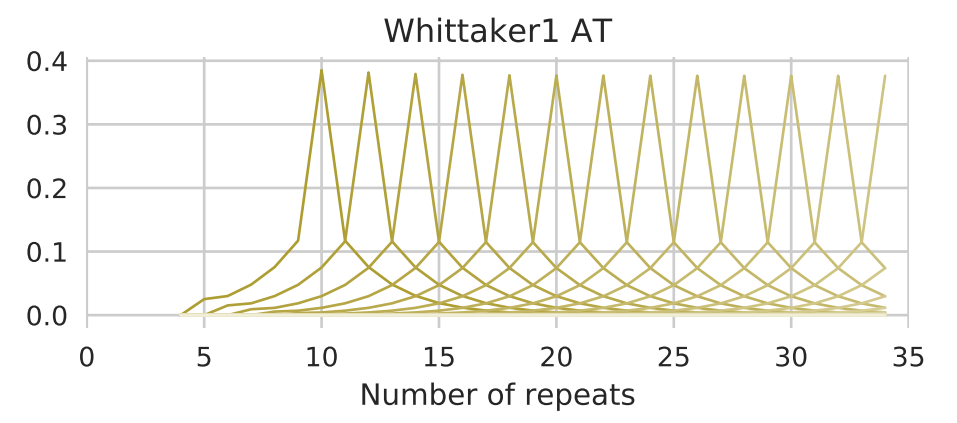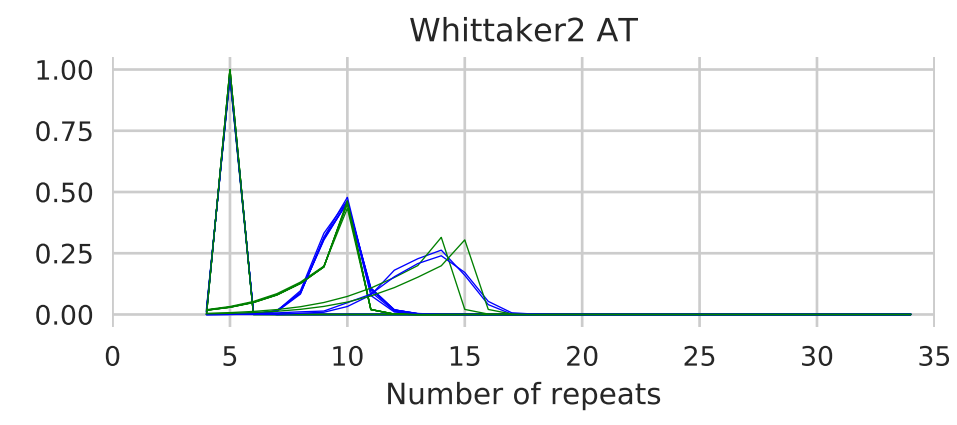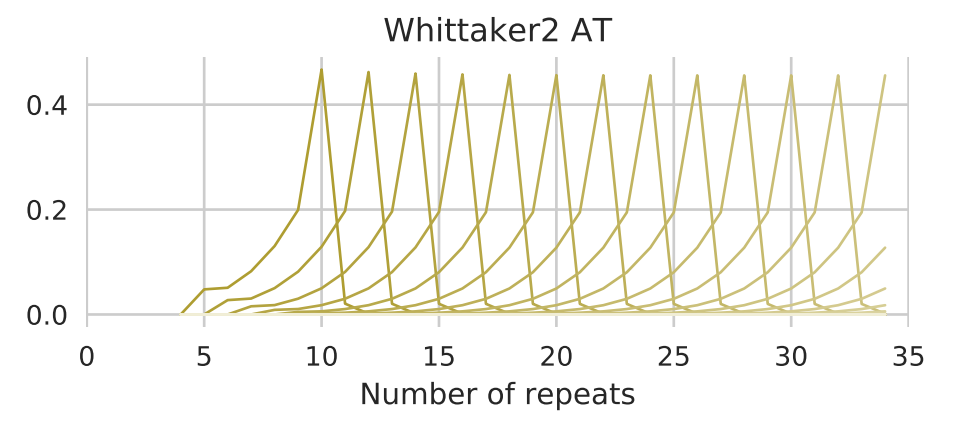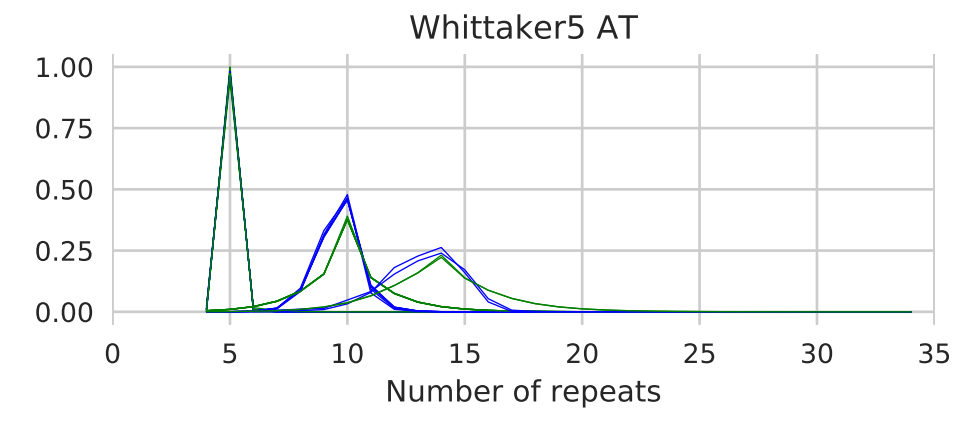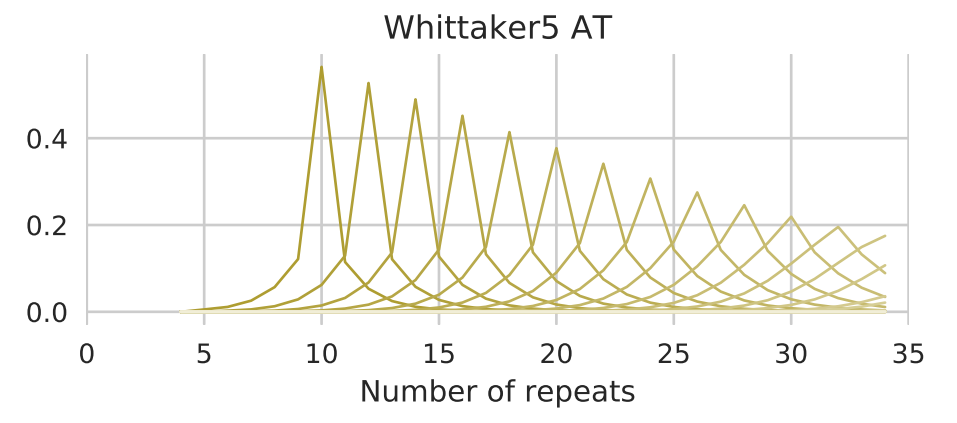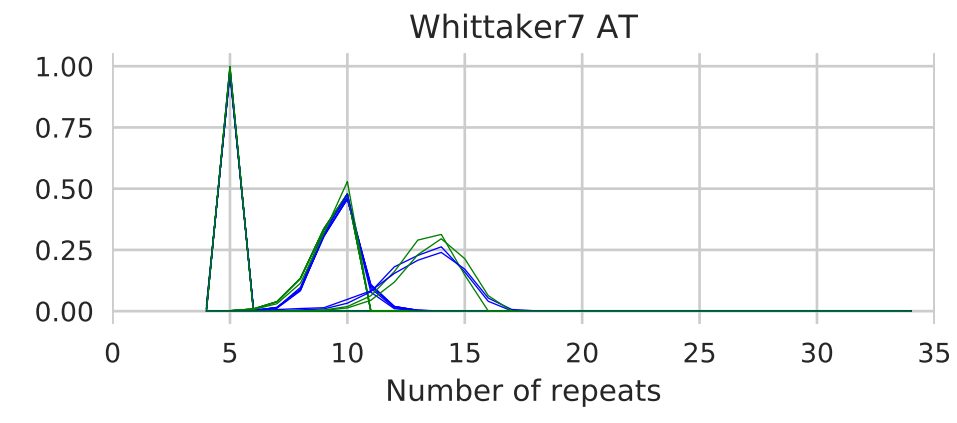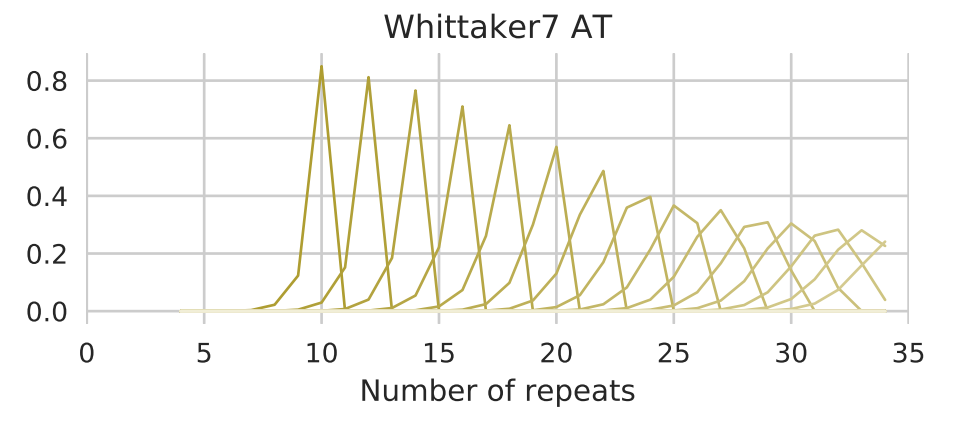

Supplement: Supplementary Data [file gky1318_supplemental_files.zip › Supplemental_Figure_S8_AT_series.pdf]

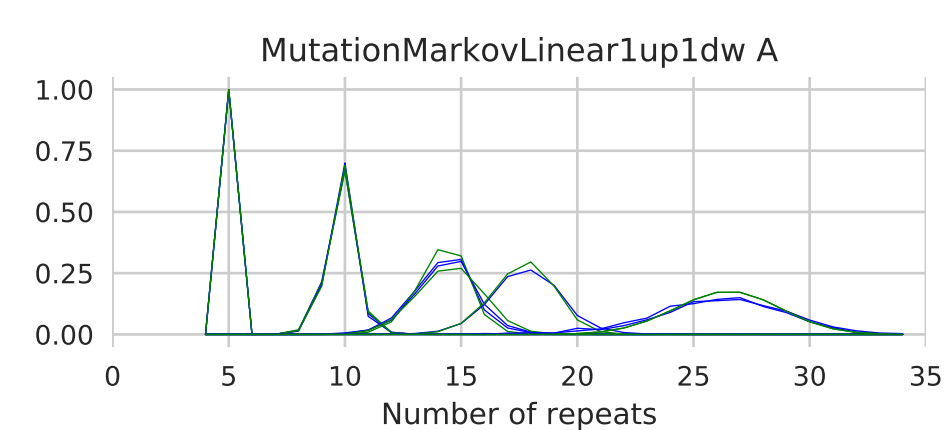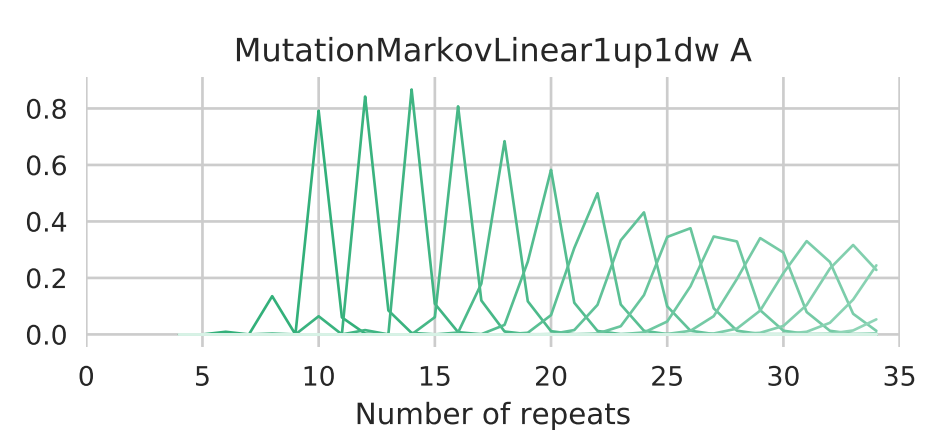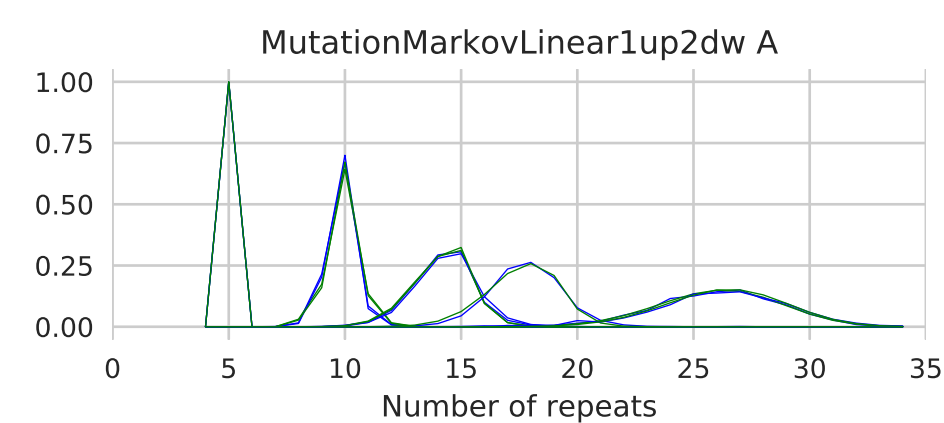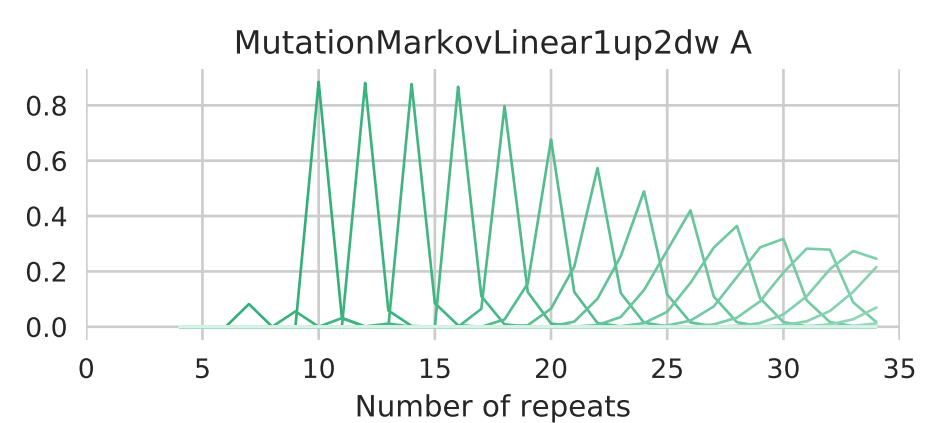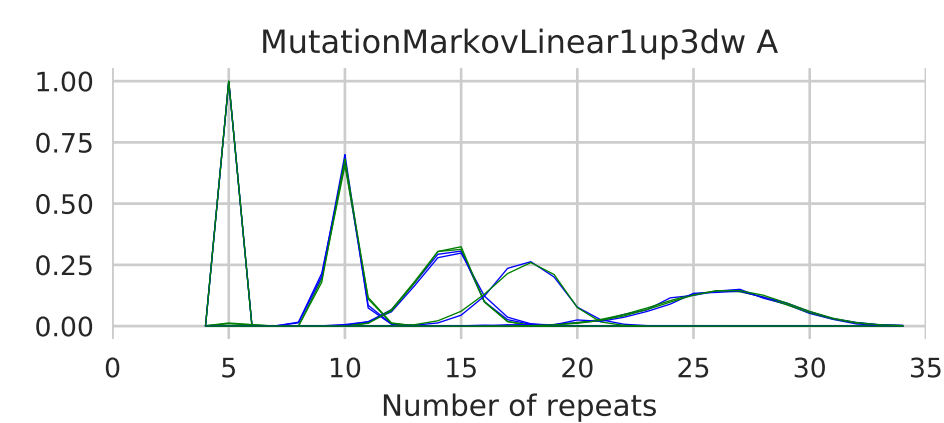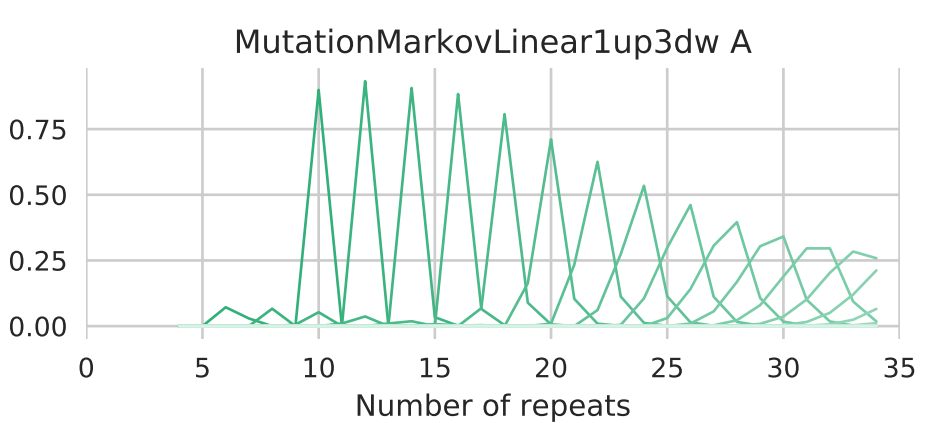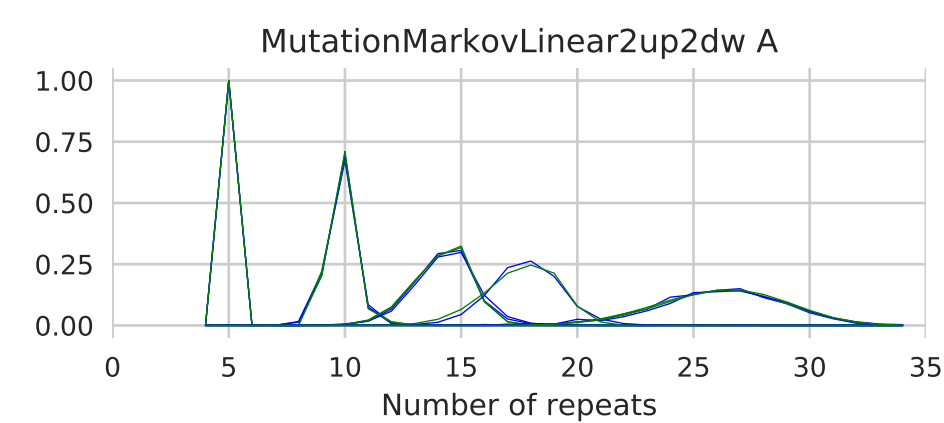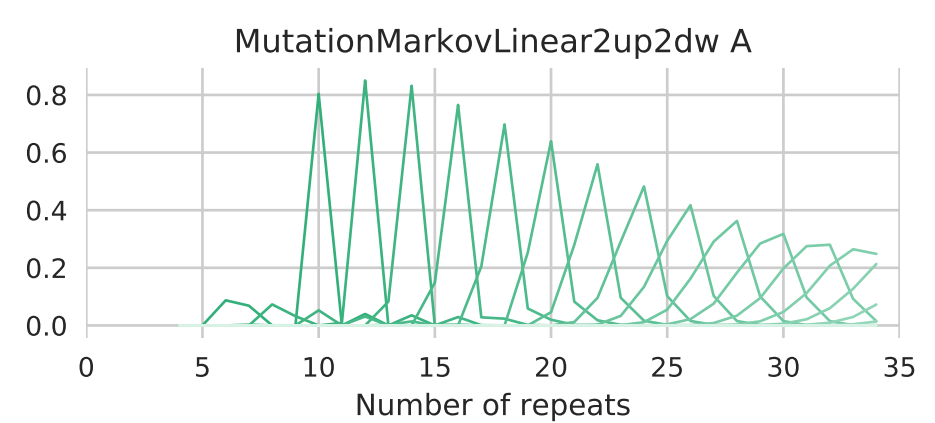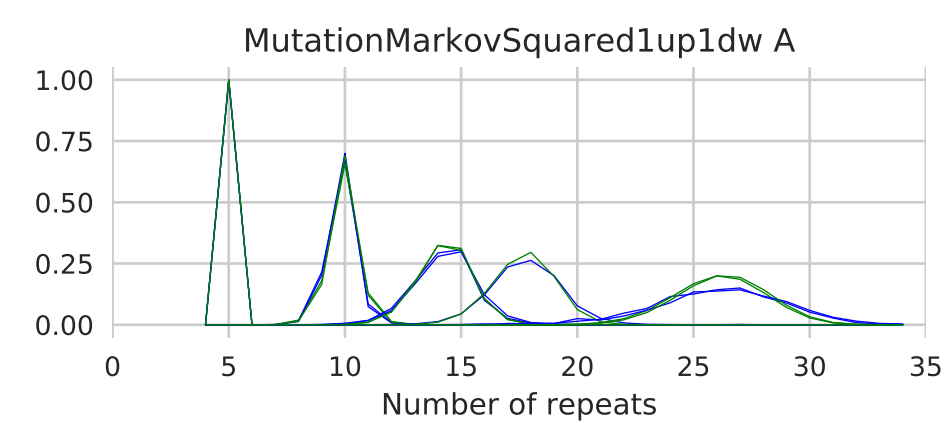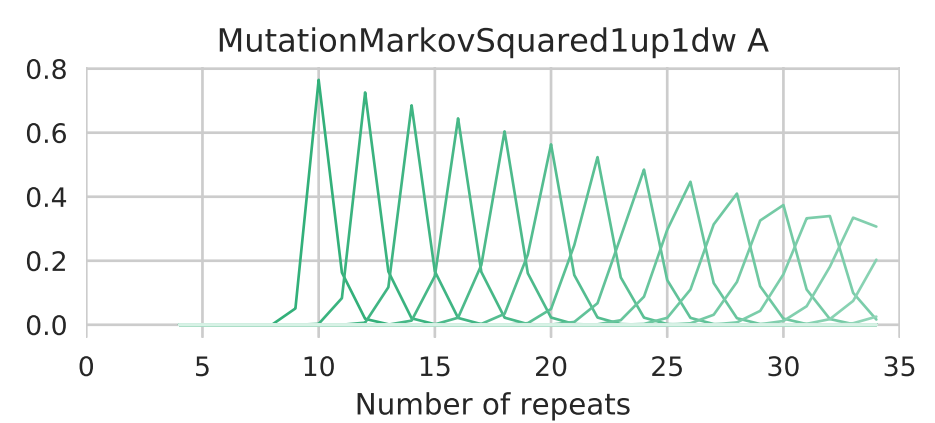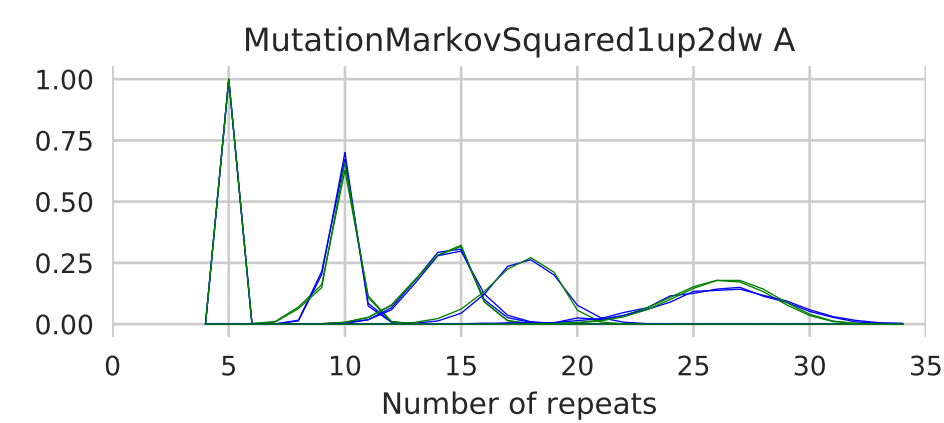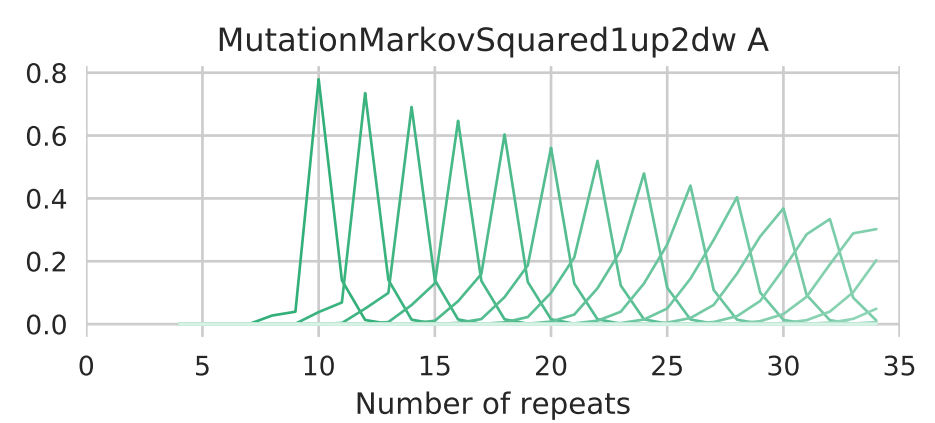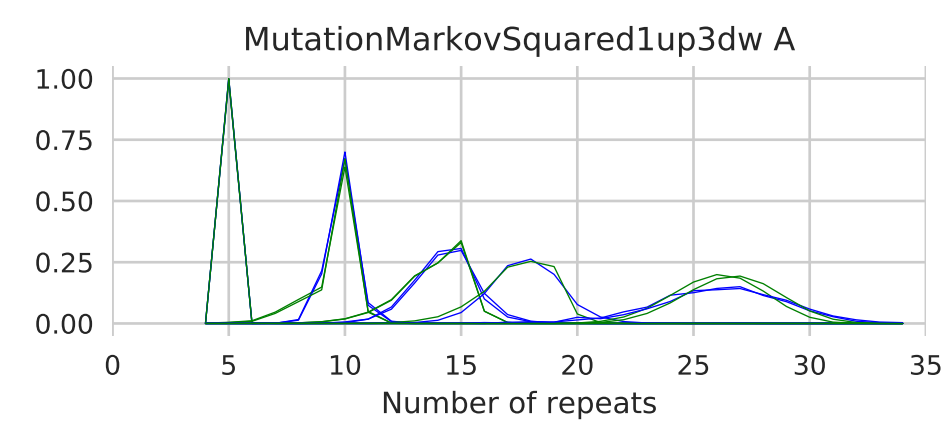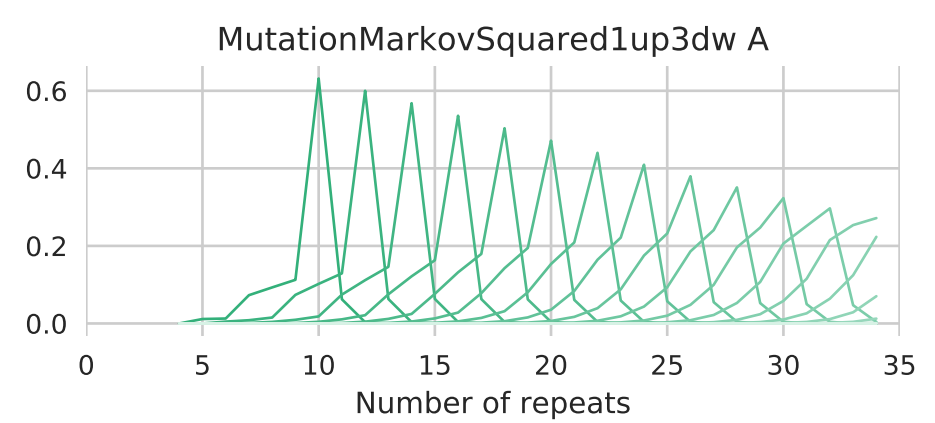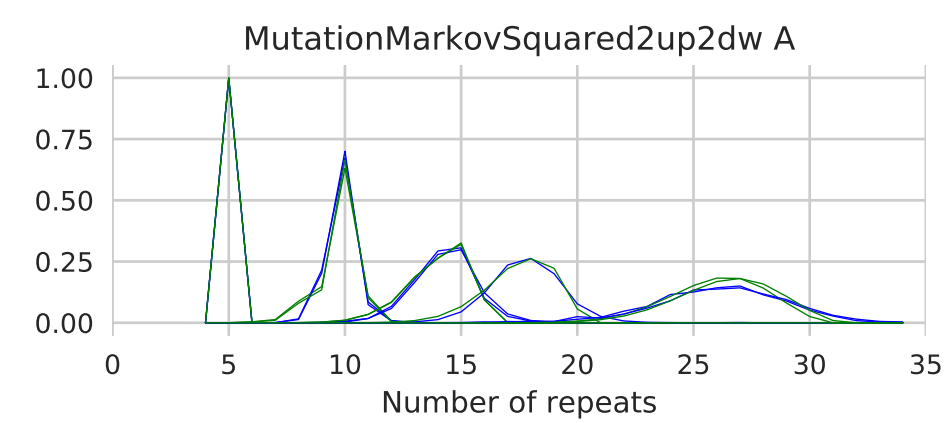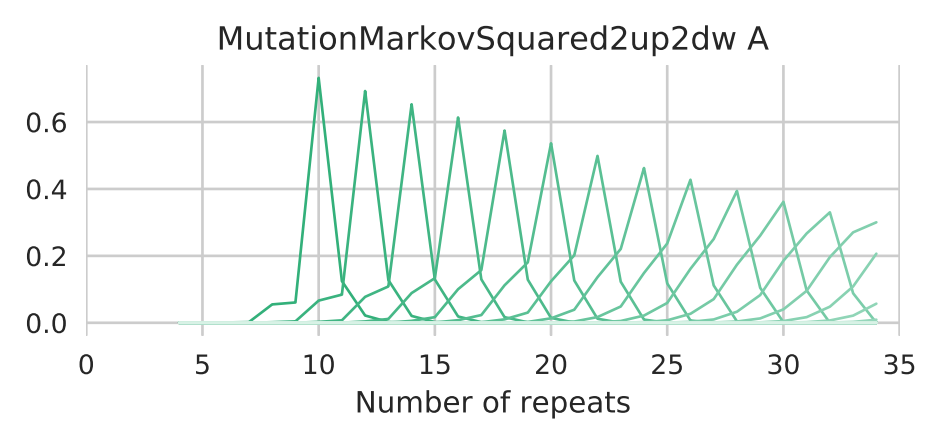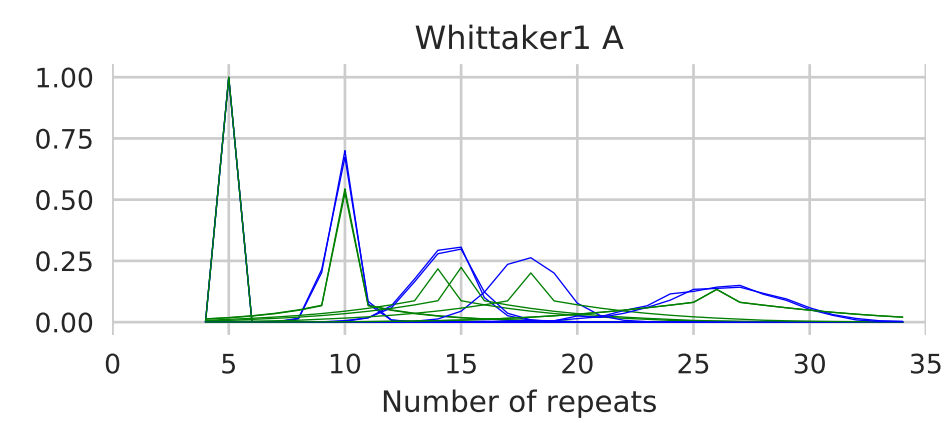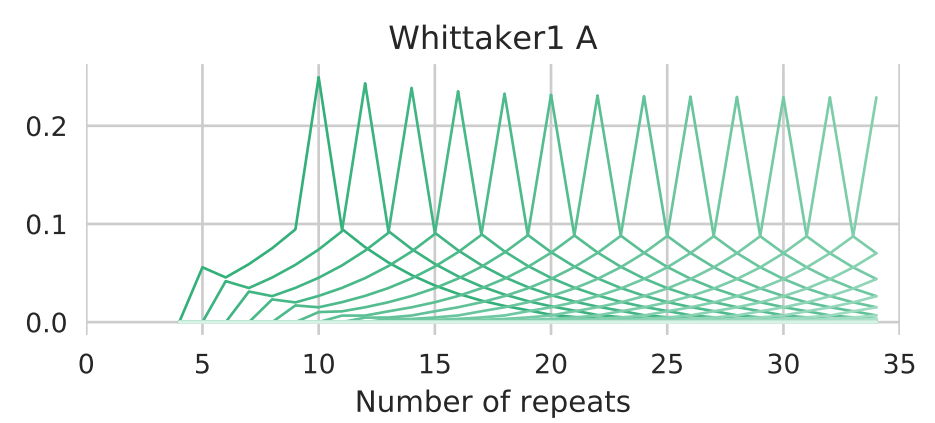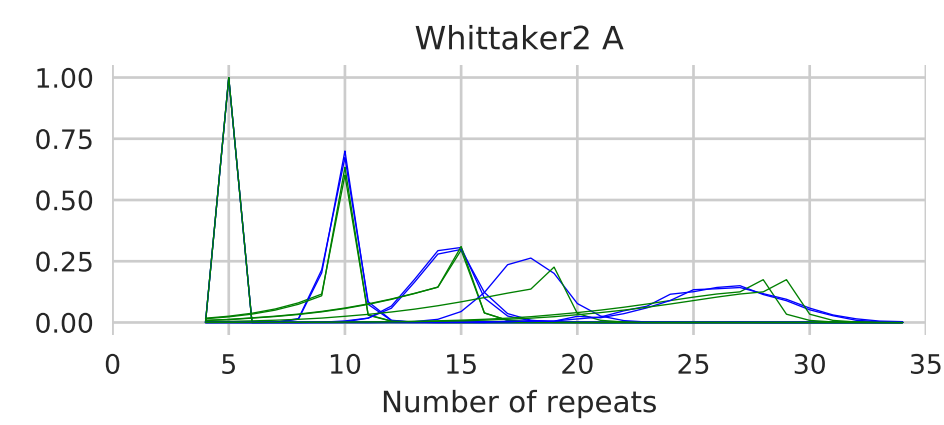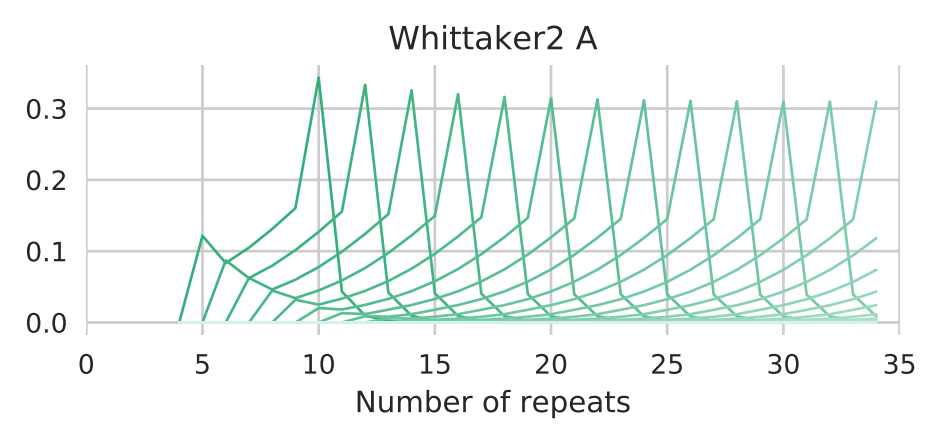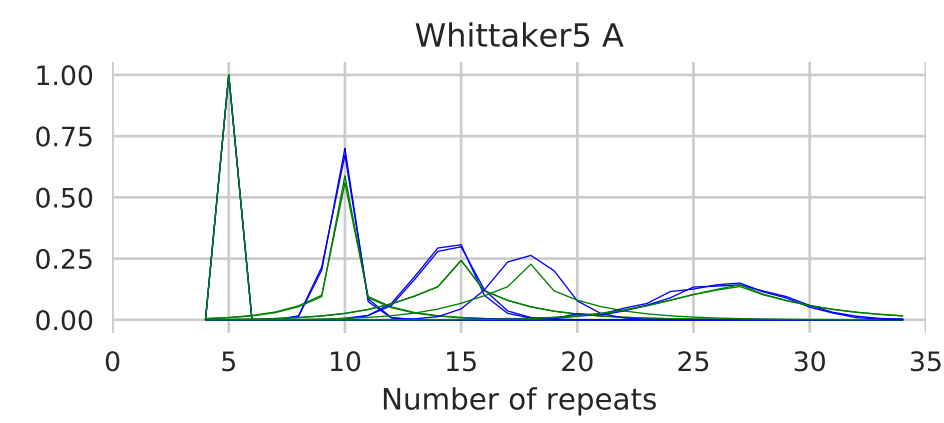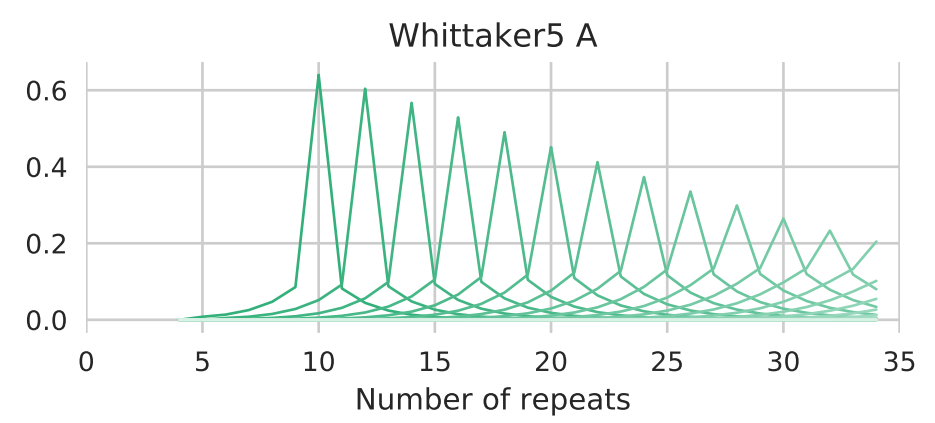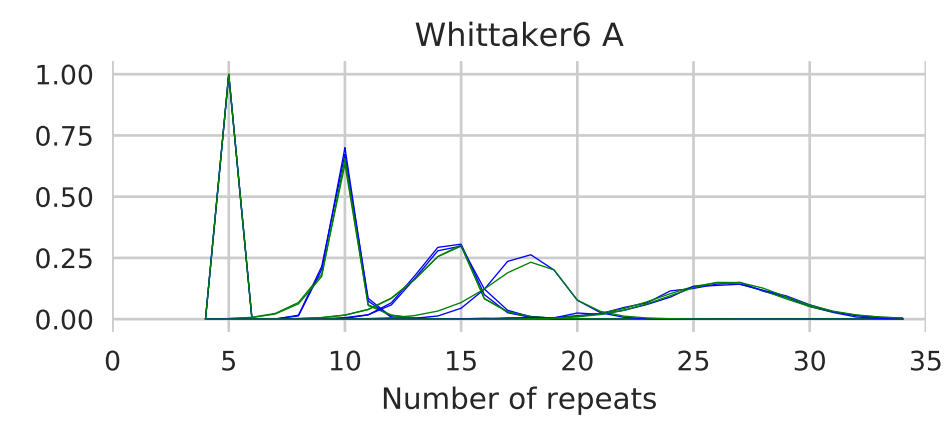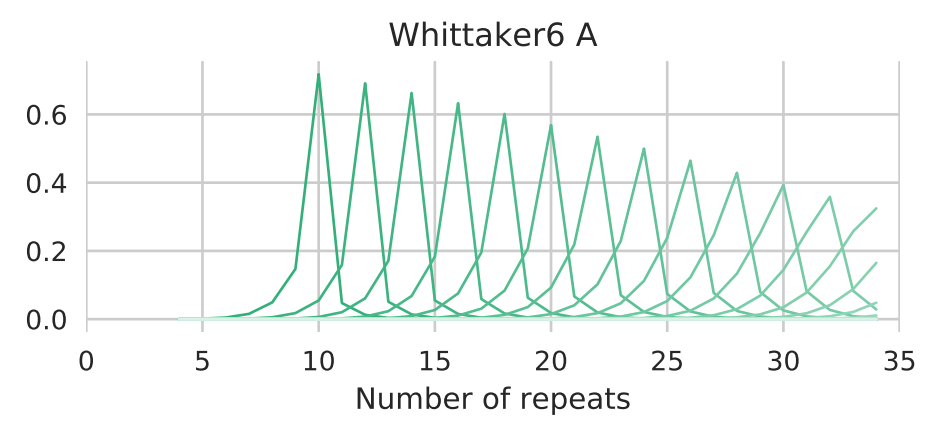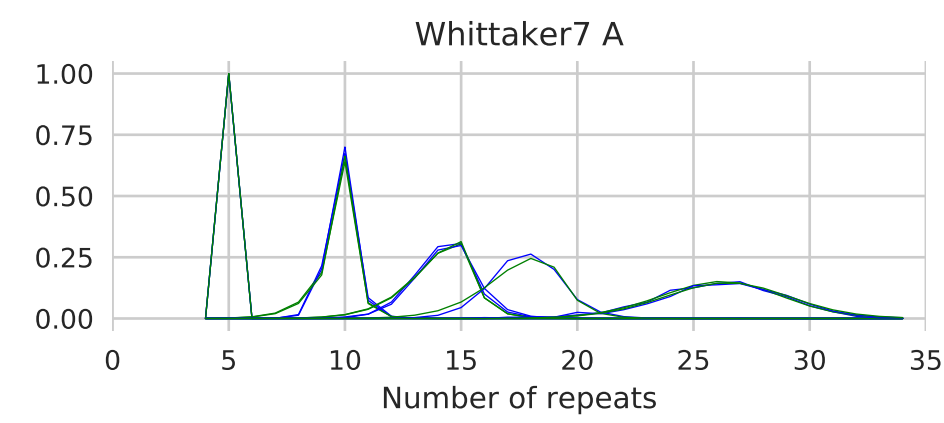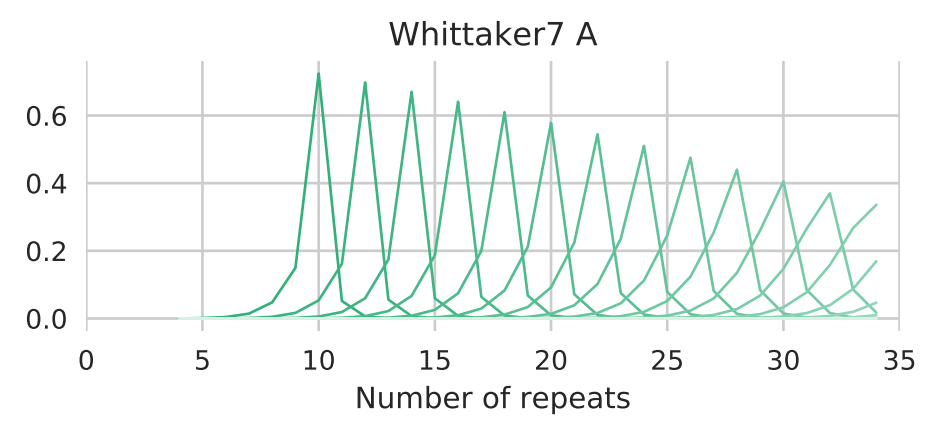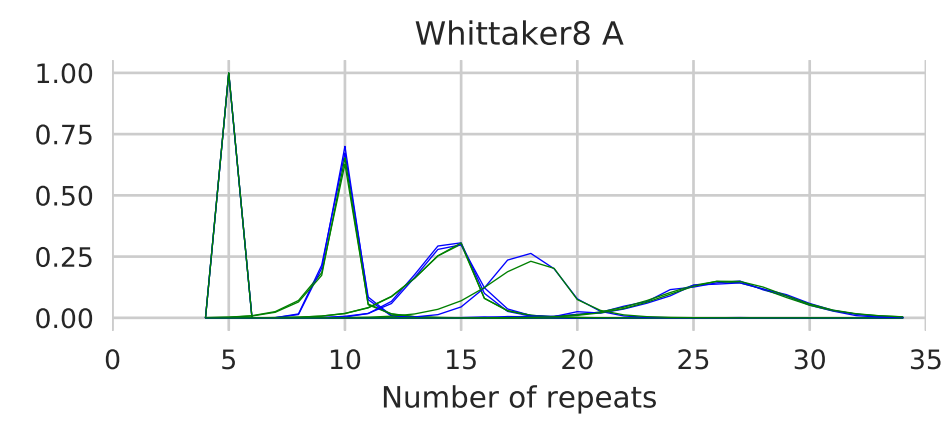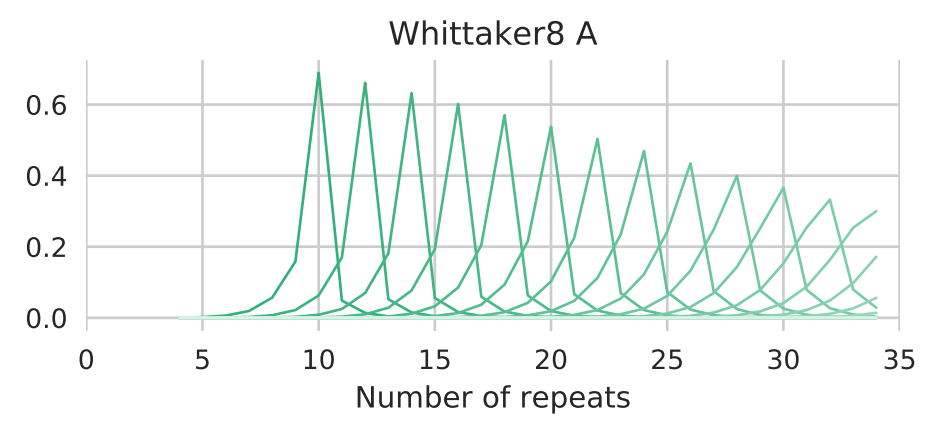

Supplement: Supplementary Data [file gky1318_supplemental_files.zip › Supplemental_Figure_S9_A_series.pdf]
